# Long-term colorectal cancer incidence in a post-endoscopic screening cohort, accounting for surveillance, by baseline polyp group, anatomic subsite, and sex

**DOI:** 10.1177/09691413251316442

**Published:** 2025-01-28

**Authors:** Emma C Robbins, Kate Wooldrage, Brian P Saunders, Amanda J Cross

**Affiliations:** 1 Cancer Screening and Prevention Research Group (CSPRG), Department of Surgery and Cancer, 4615 Imperial College London, London, UK; 2 Wolfson Unit for Endoscopy, St Mark's Hospital, London, UK

**Keywords:** Colorectal cancer, polyps, screening, surveillance

## Abstract

**Objectives:**

Colonoscopy surveillance is often performed in post-polypectomy cohorts, likely altering colorectal cancer (CRC) outcomes, but this is often not addressed in CRC incidence analyses. We examined CRC incidence post-endoscopic screening, accounting for surveillance.

**Methods:**

We examined UK Flexible Sigmoidoscopy Screening Trial participants who had no, low-risk, or high-risk (≥10 mm, ≥3 adenomas, adenomas with villous features/high-grade dysplasia) distal polyps at screening. Participants with high-risk polyps had an index colonoscopy and 81% had ≥1 surveillance colonoscopies post-screening; <1% of those with no/low-risk polyps had an index or surveillance colonoscopy. We examined CRC incidence over 21 years by anatomic subsite and sex. Standardised incidence ratios (SIRs) compared incidence to general population incidence.

**Results:**

Of 39,417 participants, 29,792 (76%), 8162 (21%), and 1463 (4%) had no, low-risk, and high-risk polyps, respectively. In the high-risk group, all-site CRC incidence was non-significantly different from that in the general population, when including all participants, just those who attended surveillance, or just those who did not attend surveillance (SIRs: 0.81 [95% confidence interval: 0.60–1.07]; 0.75 [0.54–1.03]; 1.12 [0.56–2.01], respectively). Without surveillance, compared to the general population, distal cancer incidence was lower among women and men without polyps (SIRs: 0.30 [0.24–0.37]; 0.24 [0.20–0.29], respectively) and women and men with low-risk polyps (SIRs: 0.52 [0.34–0.76]; 0.27 [0.19–0.37], respectively); proximal cancer incidence was lower among men without polyps (SIR: 0.75 [0.64–0.88]), non-significantly different among women without polyps (SIR: 1.07 [0.93–1.22]) and men with low-risk polyps (SIR: 1.22 [0.98–1.51]), but higher among women with low-risk polyps (SIR: 2.22 [1.77–2.76]).

**Conclusions:**

Women with low-risk distal polyps at flexible sigmoidoscopy screening had double the risk of proximal colon cancer, compared to the general population.

## Introduction

Endoscopic examinations can detect colorectal cancer (CRC) at early stages and prevent CRC by removing colorectal polyps (CRC precursors).^1^ People remaining at increased CRC risk post-polypectomy are recommended colonoscopy surveillance. The need for surveillance is best assessed by comparing post-polypectomy CRC risk in individuals not undergoing surveillance with that in the general population, while the benefit of surveillance is best assessed by comparing post-polypectomy CRC risk in individuals undergoing surveillance with that in the general population.^2^ Studies examining long-term post-polypectomy CRC outcomes were incorporated into the 2020 UK, US, and European surveillance guidelines^2–13^; however, many patients in these studies underwent surveillance, which likely altered CRC outcomes, but this was often not considered in analyses.

In the UK Flexible Sigmoidoscopy Screening Trial (UKFSST), ∼41,000 participants underwent flexible sigmoidoscopy (FS) screening^14–17^; there is now 21 years’ follow-up, during which only 3% of participants had surveillance. This provided a unique opportunity to examine long-term CRC incidence post-screening by baseline polyp group, anatomic subsite, and sex, accounting for effects of surveillance.

## Patients and methods

The UKFSST recruited individuals aged 55–64 years across 14 UK centres from 1994 to 1999. Exclusions are detailed elsewhere.^14–17^ Individuals were randomised (1:2) to the intervention arm (*n* = 57,237), who were offered a once-only FS screen, or control arm (*n* = 113,195), who were not contacted. This analysis included individuals screened in the trial.

The trial protocol stipulated that participants suspected to have an adenoma or hyperplastic polyp (HP) ≥ 10 mm, ≥3 adenomas, an adenoma with villous/tubulovillous histology or high-grade dysplasia, ≥20 HPs above the distal rectum, or carcinoma at FS be referred for colonoscopy, and that subsequent referral for surveillance or discharge be based on whether or not such polyps were confirmed after colonoscopy.^18^ The protocol stipulated that surveillance should involve ≥2 colonoscopies at 3-yearly intervals. Participants identified at FS to have polyps not meeting the high-risk criteria or no polyps were to be discharged without referral for colonoscopy or surveillance.^18^

We grouped baseline examinations into the ‘baseline visit’, including initial FS examinations and any completion examinations performed to fully inspect the distal colorectum and/or remove polyps. For participants who underwent colonoscopy at baseline, the baseline visit also included their initial colonoscopy and any completion examinations, collectively called their ‘index colonoscopy’.

We excluded participants who were initially screened by colonoscopy rather than by FS and those screened during the initial months of screening at one centre where the pathologist was over-diagnosing adenomas. We excluded participants who met the criteria for colonoscopy but did not undergo colonoscopy, those with extended baseline visits (>11 months), baseline CRC diagnosis or colectomy, or CRC reported within ≤11 months post-baseline, and those who may have had inflammatory bowel disease, polyposis, or Lynch syndrome (formerly hereditary non-polyposis CRC) based on endoscopist comments in baseline endoscopy reports.

We examined participants in three groups: those with high-risk polyps (adenomas or HPs ≥10 mm, ≥3 adenomas, adenomas with villous/tubulovillous histology or high-grade dysplasia), low-risk polyps not meeting the high-risk criteria, or no polyps found in the distal colorectum at baseline.

Data on baseline examinations and surveillance examinations, available through 2012, were collected from the trial centres. Cancer, death, and emigration data were obtained from cancer registries, the National Health Service (NHS) Central Register, NHS England (formerly NHS Digital), National Services Scotland, and the Office for National Statistics, available through 2017 (Wales), 2018 (Scotland), or until September 2019 (England). The National Data Opt-Out programme in England allows individuals to opt out of having their data used for research; we are not permitted to identify those who have opted out. Deaths and cancers occurring among opt-outs were excluded from data extracts provided by NHS England and their data were censored at end of follow-up.^17^

We grouped surveillance examinations into visits, comprising a single examination or an initial examination followed by completion examination(s) performed within ∼11 months of each other. To be included as surveillance, ≥1 colonoscopy or FS must have been performed at the visit. Visits reported by endoscopists as being for symptom investigation were not included as surveillance (occurring in 22 participants). Visits at which CRC was diagnosed were included as surveillance (occurring in 10 participants).

The primary outcome was incident colorectal adenocarcinoma, including cancers with unspecified morphology located between the rectum and caecum, but not those around the anus (likely squamous cell carcinomas). We stratified by subsite (distal: rectum–sigmoid colon; proximal: descending colon–caecum) for the ‘no polyps’ and low-risk groups, focussing our discussion on these subsite-specific analyses as they provided more nuanced insights than analyses of all-site CRC incidence. It was not possible to stratify by subsite for the high-risk group due to limited CRC cases. We counted traditional adenomas, serrated adenomas, and mixed hyperplastic-adenomatous polyps as adenomas, and considered HPs separately, reflecting polyp classification during the screening period.

### Statistical analysis

We examined baseline patient, procedural, and polyp characteristics, comparing across polyp groups and sex using χ^2^ tests. For the high-risk group, we compared baseline characteristics between those with and without surveillance using χ^2^ tests.

We examined CRC incidence, with time-at-risk starting from the latest baseline examination, censoring at first CRC diagnosis, emigration, death, or end of follow-up. We considered the earliest diagnosed CRC only and counted distal and proximal cancers in participants with synchronous cancers. For the ‘no polyps’ and low-risk groups, we estimated CRC incidence without surveillance, censoring at any first surveillance visit. For the high-risk group, we estimated CRC incidence (a) among all participants, including those who attended surveillance and those who did not, (b) among just those who attended surveillance, and (c) among just those who did not attend surveillance. We did not compare CRC incidence among those who attended surveillance and those who did not because the difference in exposure to surveillance would have differentially altered CRC incidence in these two subgroups. All incidence analyses were stratified by sex for the ‘no polyps’ and low-risk groups but not the high-risk group due to limited CRC cases.

We used Kaplan–Meier analyses to show time to CRC diagnosis and estimate cumulative incidence at 10 and 20 years after baseline. We compared CRC incidence to that in the general population with standardised incidence ratios (SIRs) and exact Poisson 95% confidence intervals (CIs). We calculated SIRs by dividing observed CRC cases by expected cases, estimated by multiplying 5-year age group- and sex-specific person-years at risk with corresponding CRC incidence in the general population (England, 2005).^19^ Using 2005 data allowed comparisons with general population incidence prior to the introduction of the Bowel Cancer Screening Programme (BCSP).^20^ We examined SIRs by baseline characteristics in the ‘no polyps’ and low-risk groups to identify recurring patterns.

We compared CRC incidence between the ‘no polyps’ and low-risk groups, using log-rank tests to compare cumulative incidence curves and Cox models to estimate hazard ratios (HRs) with 95%CIs; multivariable models were adjusted for baseline age group and characteristics independently associated with CRC incidence in the two polyp groups combined. We examined variation in the HRs comparing CRC incidence between the two polyp groups by baseline age group and endoscopist adenoma detection rate (ADR) ranking (high, intermediate, or low^21,22^) using interaction terms. Participants with high-risk polyps were not included in these comparisons because CRC incidence would have been differentially altered as they all had an index colonoscopy, compared to 0.6% of ‘no polyps’/low-risk participants.

The UKFSST is registered (ISRCTN:28352761; https://www.isrctn.com/ISRCTN28352761) and the protocol is online.^18^ We conducted analyses in Stata/IC V.17 (Statacorp. 2021. Stata Statistical Software: Release 17. College Station, Texas: Statacorp LLC). We set alpha at 0.05.

## Results

**Figure 1.**
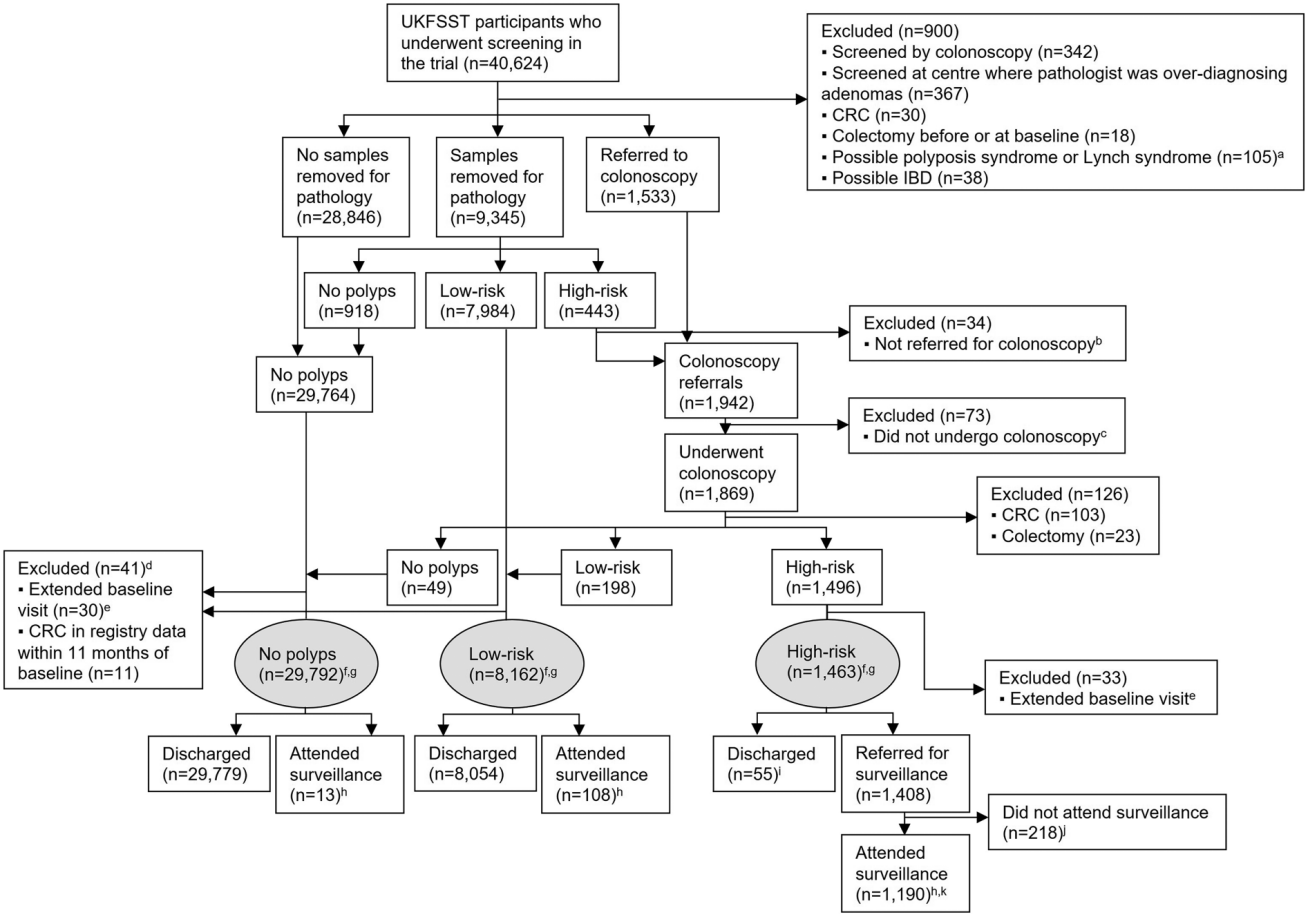
Flow diagram of the selection of the study population (*n* = 39,417). ^a^In the majority of these participants (87%, *n* = 91), ≥20 HPs above the distal rectum were reported. In the remaining participants, ≥10 adenomas or possible Lynch syndrome (formerly Hereditary Nonpolyposis CRC) were reported. ^b^These participants were classified and treated as being at low-risk in the trial. However, for the present analysis, they were considered to be at high-risk owing to a pathologically confirmed HP ≥10 mm, adenoma ≥10 mm, or adenoma with tubulovillous histology; since they were not referred for colonoscopy, they were excluded from analysis. ^c^The majority of these participants (75%, *n* = 55) refused colonoscopy. For the remaining participants, reasons for not undergoing colonoscopy included: died, moved away, too ill, colonoscopy cancelled, other, unknown. ^d^21 participants were from the ‘no polyps’ group and 20 participants were from the low-risk group. ^e^A baseline visit that spanned >11 months. ^f^Participants in the high-risk group had an adenoma or HP ≥10 mm, ≥3 adenomas, or an adenoma with villous/tubulovillous histology or high-grade dysplasia at baseline. Participants in the low-risk group had polyps detected at baseline that did not meet the high-risk criteria. Participants in the ‘no polyps’ group had no polyps detected at baseline. ^g^Median follow-up in the ‘no polyps’, low-risk, and high-risk groups was 21 years (IQR: 19–22), 21 years (IQR: 17–22), and 20 years (IQR: 16–21), respectively. ^h^Attended ≥1 surveillance visits during follow-up, with surveillance visits comprising ≥1 colonoscopy or flexible sigmoidoscopy. Most surveillance visits (97%) comprised ≥1 colonoscopy, while the remainder (3%) comprised ≥1 flexible sigmoidoscopy with no colonoscopy. ^i^The majority of these participants were discharged because they did not attend all scheduled baseline examinations (16%, *n* = 9) or for unknown reasons (67%, *n* = 37). For the remaining participants, reasons for being discharged included: too ill, difficult index colonoscopy, patient non-cooperation, endoscopist considered polyps to be low-risk, died. ^j^The majority of these participants refused surveillance (60%, *n* = 131) or had died (13%, *n* = 28) or moved away (13%, *n* = 28). For the remaining participants, reasons for not undergoing surveillance included: too ill, did not attend, patient cancelled, only follow-up visit performed to investigate symptoms, other, unknown. ^k^Among the 1190 participants in the high-risk group who attended surveillance, 400 (34%) had one surveillance visit, 500 (42%) had two surveillance visits, and 290 (24%) had three or more surveillance visits. The median time interval from baseline to first surveillance was three years. CRC: colorectal cancer; HP: hyperplastic polyp; IBD: inflammatory bowel disease; UKFSST: UK Flexible Sigmoidoscopy Screening Trial; IQR: interquartile range.

After exclusions, 39,417 participants were included in the analysis: 29,792 (76%: women, *n* = 16,059; men, *n* = 13,733) were in the ‘no polyps’ group, 8162 (21%: women, *n* = 3121; men, *n* = 5041) in the low-risk group, and 1463 (4%: women, *n* = 449; men, *n* = 1014) in the high-risk group (Figure 1, Supplemental material Table 1). Among the ‘no polyps’, low-risk, and high-risk groups, 13 (0.04%), 108 (1.3%), and 1190 (81%) had surveillance during follow-up, respectively (Figure 1).

Overall, a greater proportion of women had no polyps (82% vs 69%) and a smaller proportion had an index colonoscopy (3% vs 6%), compared to men. Participants with no polyps were more likely to have been screened by endoscopists in the low ADR ranking group (low-detectors), compared to those identified with low-risk or high-risk polyps (Supplemental material Table 1). In each polyp group, women were more likely than men to have been screened by high-detectors or had incomplete baseline examinations. In the ‘no polyps’ and high-risk groups, women were more likely than men to have excellent bowel preparation at baseline. Among low-risk participants, men were more prone to having adenomas, larger (5–9 mm) adenomas, more or larger HPs, HPs not confined to the rectum/rectosigmoid, or both adenomas and HPs, compared to women. Among high-risk participants, men tended to have more adenomas and HPs, and both adenomas and HPs, compared to women (Supplemental material Table 1). High-risk participants attending surveillance were more likely than non-attenders to have a complete index colonoscopy; polyp characteristics also differed between attenders and non-attenders (Supplemental material Table 2).

Over a median follow-up of 21 years (interquartile range: 19–22), 302, 116, and 9 CRCs were diagnosed among women in the ‘no polyps’, low-risk, and high-risk groups, respectively, and 269, 133, and 41 CRCs were diagnosed among men in each respective group (Supplemental material Table 3).

**Figure 2.**
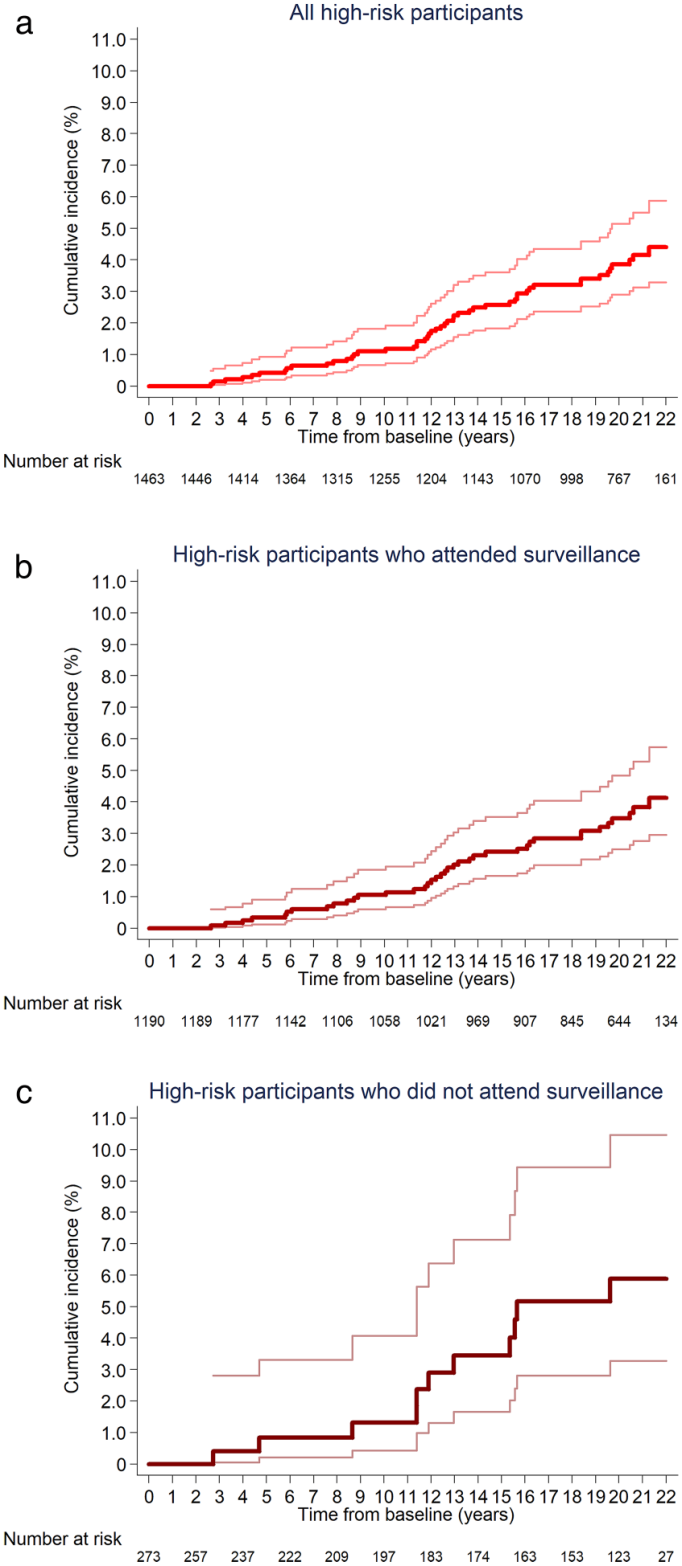
Kaplan–Meier estimates of cumulative all-site CRC incidence in the high-risk group, among all high-risk participants (a), those who attended surveillance (b), and those who did not attend surveillance (c) (*n* = 1463). 95% confidence intervals are shown for each curve. Participants in the high-risk group had an adenoma or HP ≥10 mm, ≥3 adenomas, or an adenoma with villous/tubulovillous histology or high-grade dysplasia at baseline. (a) A total of 50 cases were included in the count for cumulative all-site CRC incidence during follow-up among all high-risk participants, including those who attended one or more surveillance visits and those who did not. (b) A total of 39 cases were included in the count for cumulative all-site CRC incidence during follow-up among high-risk participants who attended one or more surveillance visits. (c) A total of 11 cases were included in the count for cumulative all-site CRC incidence during follow-up among high-risk participants who did not attend surveillance. CRC: colorectal cancer; HP: hyperplastic polyp.

Compared to the general population, all-site CRC incidence was not statistically significantly different in the high-risk group, when including all high-risk participants (SIR: 0.81 [95%CI 0.60–1.07]) and when restricting to those who attended surveillance (SIR: 0.75 [0.54–1.03]) or those who did not attend surveillance (SIR: 1.12 [0.56–2.01]). However, SIR point estimates were below one and the upper bounds of the SIR 95%CIs close to one for all high-risk participants and for those who attended surveillance. Additionally, it is worth noting that there were only 4153 person-years and 11 CRC cases in analyses of those who did not attend surveillance, resulting in a wide SIR 95%CI (Table 1). Among all high-risk participants, cumulative all-site CRC incidence was 1.1% (95%CI 0.7–1.8) and 3.9% (2.9–5.1) at 10 and 20 years after baseline, respectively. Corresponding figures were 1.1% (0.6–1.8) and 3.5% (2.5–4.8), respectively, among those who attended surveillance, and 1.3% (0.4–4.1) and 5.9% (3.3–10.5) among those who did not attend surveillance (Table 1). Cumulative CRC incidence is illustrated in Figure 2.

**Table 1.** All-site colorectal cancer incidence and age-sex-SIRs in the high-risk group (*n* = 1463).

High-risk participants included in analysis^a^	Total no. of cases	Incidence rate per 100,000 person-years (95%CI)	Follow-up				Standardisation	
			10 years		20 years			
			No. of cases	Cumulative incidence, % (95%CI)	No. of cases	Cumulative incidence, % (95%CI)	No. of expected cases	SIR (95%CI)
All high-risk participants (*n* = 1463)^b^	50	194 (147–256)	15	1.1 (0.7–1.8)	46	3.9 (2.9–5.1)	62	0.81 (0.60–1.07)
Those who attended ≥1 surveillance visits (*n* = 1190)^b^	39	180 (132–247)	12	1.1 (0.6–1.8)	35	3.5 (2.5–4.8)	52	0.75 (0.54–1.03)
Those who did not attend surveillance (*n* = 273)^b^	11	265 (147–478)	3	1.3 (0.4–4.1)	11	5.9 (3.3–10.5)	10	1.12 (0.56–2.01)

CI: confidence interval; SIR: standardised incidence ratio; HP: hyperplastic polyp.

^a^ Participants in the high-risk group had an adenoma or HP ≥10 mm, ≥3 adenomas, or an adenoma with villous/tubulovillous histology or high-grade dysplasia at baseline.

^b^ Each participant's follow-up time was included from start of time-at-risk and censored at end of follow-up; in total, there were 25,780 person-years in the analysis including all high-risk participants, 21,627 person-years in the analysis including just those high-risk participants who attended one or more surveillance visits, and 4153 person-years in the analysis including just those high-risk participants who did not attend surveillance.

The following results for the ‘no polyps’ and low-risk groups are in the absence of surveillance. Compared to the general population, distal cancer incidence was substantially lower among women and men without polyps (SIRs: 0.30 [95%CI 0.24–0.37] and 0.24 [0.20–0.29], respectively) and among women and men with low-risk polyps (SIR: 0.52 [0.34–0.76] and 0.27 [0.19–0.37], respectively) (Table 2). Compared to the general population, proximal cancer incidence was lower among men without polyps (SIR: 0.75 [0.64–0.88]) and not statistically significantly different among women without polyps (SIR: 1.07 [0.93–1.22]) or men with low-risk polyps (SIR: 1.22 [0.98–1.51]), although the point estimate of the latter SIR was above one and the lower bound of the SIR 95%CI close to one. Proximal cancer incidence was substantially higher among women with low-risk polyps than in the general population (SIR: 2.22 [1.77–2.76]) (Table 2). Compared to the general population, all-site CRC incidence was lower among women without polyps (SIR: 0.58 [0.52–0.65]), men without polyps (SIR: 0.39 [0.34–0.44]), and men with low-risk polyps (SIR: 0.54 [0.45–0.64]), while it was not statistically significantly different among women with low-risk polyps (SIR: 1.17 [0.97–1.41]) (Table 2).

**Table 2.** Distal, proximal, and all-site colorectal cancer incidence without surveillance and age-sex-SIRs in women and men in the ‘no polyps’ and low-risk groups (*n* = 37,954).

Subsite	Polyp group^a^, sex	Total no. of cases	Incidence rate per 100,000 person-years (95%CI)	Follow-up				Standardisation	
				10 years		20 years			
				No. of cases	Cumulative incidence, % (95%CI)	No. of cases	Cumulative incidence, % (95%CI)	No. of expected cases	SIR (95%CI)
Distal	No polyps, women (*n* = 16,059)^b^	81	26 (21–32)	20	0.1 (0.1–0.2)	75	0.5 (0.4–0.7)	270	0.30 (0.24–0.37)
	No polyps, men (*n* = 13,733)^b^	102	40 (33–49)	28	0.2 (0.2–0.3)	86	0.7 (0.6–0.9)	423	0.24 (0.20–0.29)
	Low-risk, women (*n* = 3121)^b^	26	44 (30–65)	7	0.2 (0.1–0.5)	23	0.9 (0.6–1.3)	50	0.52 (0.34–0.76)
	Low-risk, men (*n* = 5041)^b^	40	45 (33–61)	14	0.3 (0.2–0.5)	36	0.9 (0.7–1.3)	149	0.27 (0.19–0.37)
Proximal	No polyps, women (*n* = 16,059)^b^	212	68 (59–77)	60	0.4 (0.3–0.5)	184	1.3 (1.1–1.5)	198	1.07 (0.93–1.22)
	No polyps, men (*n* = 13,733)^b^	154	61 (52–71)	47	0.4 (0.3–0.5)	137	1.2 (1.0–1.4)	204	0.75 (0.64–0.88)
	Low-risk, women (*n* = 3121)^b^	82	140 (112–173)	23	0.8 (0.5–1.2)	74	2.8 (2.2–3.5)	37	2.22 (1.77–2.76)
	Low-risk, men (*n* = 5041)^b^	88	98 (80–121)	24	0.5 (0.4–0.8)	78	2.0 (1.6–2.4)	72	1.22 (0.98–1.51)
All-site^c^	No polyps, women (*n* = 16,059)^b^	302	96 (86–108)	82	0.5 (0.4–0.7)	268	1.9 (1.7–2.1)	522	0.58 (0.52–0.65)
	No polyps, men (*n* = 13,733)^b^	267^d^	105 (93–119)	77	0.6 (0.5–0.7)	233	2.0 (1.8–2.3)	687	0.39 (0.34–0.44)
	Low-risk, women (*n* = 3121)^b^	114	194 (162–233)	31	1.1 (0.7–1.5)	103	3.8 (3.2–4.6)	97	1.17 (0.97–1.41)
	Low-risk, men (*n* = 5041)^b^	130^d^	145 (122–172)	39	0.9 (0.6–1.2)	115	2.9 (2.4–3.4)	242	0.54 (0.45–0.64)

CI: confidence interval; SIR: standardised incidence ratio; CRC: colorectal cancer.

^a^ Participants in the low-risk group had polyps detected at baseline that did not meet the following high-risk criteria: an adenoma or HP ≥10 mm, ≥3 adenomas, or an adenoma with villous/tubulovillous histology or high-grade dysplasia. Participants in the ‘no polyps’ group had no polyps detected at baseline.

^b^ Each participant's follow-up time was included from start of time-at-risk and censored at any first surveillance visit; in total, there were 313,279, 253,445, 58,710, and 89,543 person-years in the analyses including women with no polyps, men with no polyps, women with low-risk polyps, and men with low-risk polyps, respectively.

^c^ For all-site CRC incidence, participants diagnosed with distal cancer, proximal cancer, or CRC for which the site was unspecified were included in the counts of CRC cases. There were nine, 13, six, and three participants with a CRC for which the site was unspecified among women in the ‘no polyps’ group, men in the ‘no polyps’ group, women in the low-risk group, and men in the low-risk group, respectively.

^d^ Among men in the ‘no polyps’ group, 269 CRCs were diagnosed in 267 men because two men had synchronous distal and proximal cancers. Among men in the low-risk group, 131 CRCs were diagnosed in 130 men because one man had synchronous distal and proximal cancers.

SIRs for distal cancer tended to increase across the endoscopist ADR ranking groups in both polyp groups and sexes (Supplemental material Table 4). SIRs for proximal cancer tended to be higher for participants with adenomas or HPs 5–9 mm, compared to those with adenomas or HPs <5 mm. Among women with low-risk distal polyps, SIRs for proximal cancer were consistently ∼2-fold greater than one across baseline characteristics (Supplemental material Table 5).

**Figure 3.**
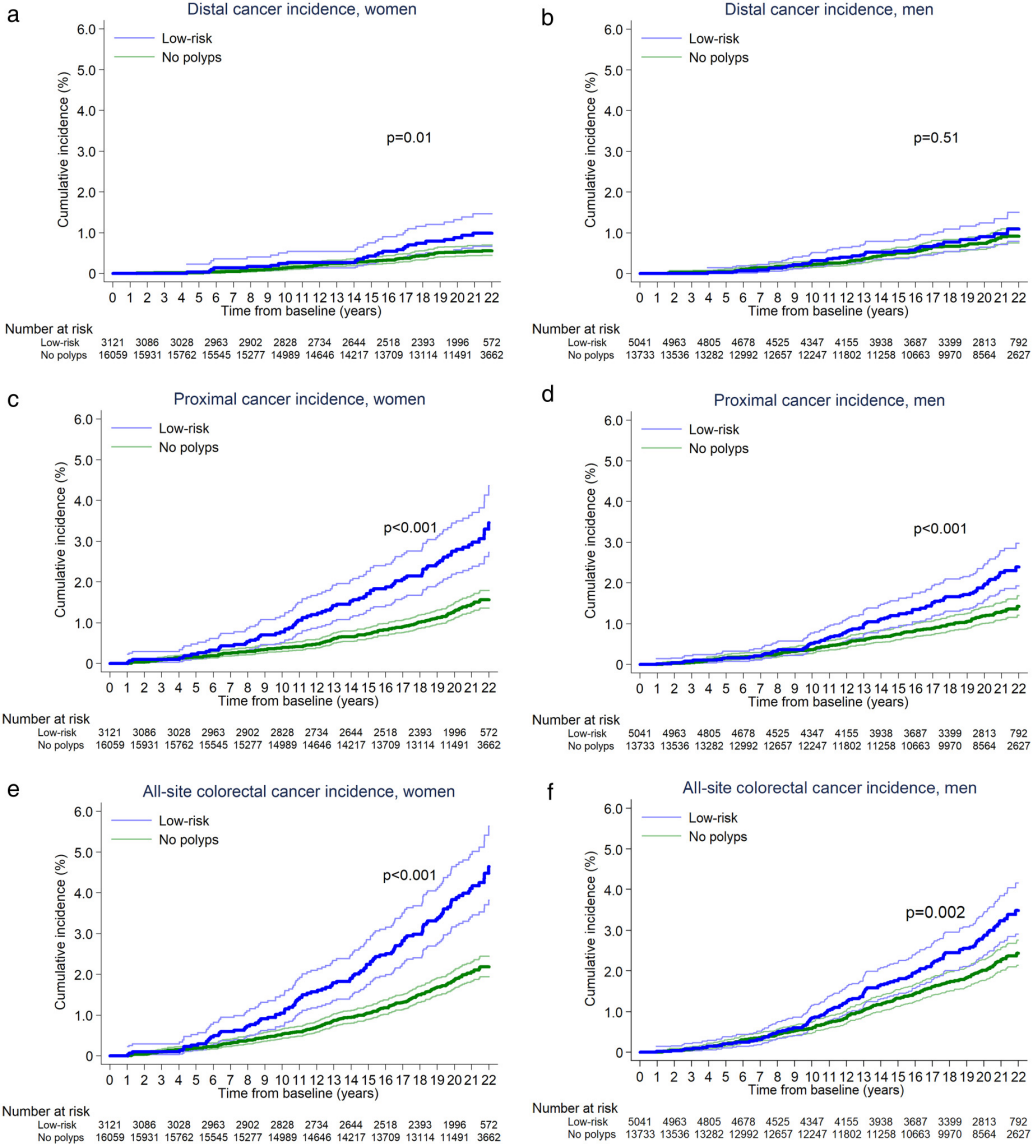
Kaplan–Meier estimates of cumulative distal (a-b), proximal (c-d), and all-site (e-f) CRC incidence without surveillance in women and men in the ‘no polyps’ and low-risk groups (*n* = 37,954). 95% confidence intervals are shown for each curve. Participants in the low-risk group had polyps detected at baseline that did not meet the following high-risk criteria: an adenoma or HP ≥10 mm, ≥3 adenomas, or an adenoma with villous/tubulovillous histology or high-grade dysplasia. Participants in the ‘no polyps’ group had no polyps detected at baseline. *p*-values shown on the graphs are for the comparison of cumulative incidence curves for the ‘no polyps’ and low-risk groups, calculated with the log-rank test. (a) In women, a total of 81 and 26 cases, respectively, were included in the counts for cumulative distal cancer incidence in the ‘no polyps’ and low-risk groups during follow-up, without surveillance. (b) In men, a total of 102 and 40 cases, respectively, were included in the counts for cumulative distal cancer incidence in the ‘no polyps’ and low-risk groups during follow-up, without surveillance. (c) In women, a total of 212 and 82 cases, respectively, were included in the counts for cumulative proximal cancer incidence in the ‘no polyps’ and low-risk groups during follow-up, without surveillance. (d) In men, a total of 154 and 88 cases, respectively, were included in the counts for cumulative proximal cancer incidence in the ‘no polyps’ and low-risk groups during follow-up, without surveillance. (e) In women, a total of 302 and 114 cases, respectively, were included in the counts for cumulative all-site CRC incidence in the ‘no polyps’ and low-risk groups during follow-up, without surveillance. (f) In men, a total of 267 and 130 cases, respectively, were included in the counts for cumulative all-site CRC incidence in the ‘no polyps’ and low-risk groups during follow-up, without surveillance. CRC: colorectal cancer; HP: hyperplastic polyp.

Distal cancer cumulative incidence curves were statistically significantly different between the ‘no polyps’ and low-risk groups in women but not men, with the curves in women lying relatively close together but showing some divergence around 15 years after baseline (Figure 3). At 10 years after baseline, cumulative distal cancer incidence was 0.1% (95%CI 0.1–0.2) and 0.2% (0.1–0.5) among women with no polyps and low-risk polyps, respectively, and 0.2% (0.2–0.3) and 0.3% (0.2–0.5) among men with no polyps and low-risk polyps, respectively. At 20 years after baseline, corresponding figures were 0.5% (0.4–0.7) and 0.9% (0.6–1.3) among women with no polyps and low-risk polyps, respectively, and 0.7% (0.6–0.9) and 0.9% (0.7–1.3) among men with no polyps and low-risk polyps, respectively (Table 2).

Proximal cancer cumulative incidence curves were substantially different between the ‘no polyps’ and low-risk groups in both sexes, with curves diverging around 5 and 10 years after baseline in women and men, respectively (Figure 3). At 10 years after baseline, cumulative proximal cancer incidence was 0.4% (95%CI 0.3–0.5) and 0.8% (0.5–1.2) among women with no polyps and low-risk polyps, respectively, and 0.4% (0.3–0.5) and 0.5% (0.4–0.8) among men with no polyps and low-risk polyps, respectively. At 20 years after baseline, corresponding figures were 1.3% (1.1–1.5) and 2.8% (2.2–3.5) among women with no polyps and low-risk polyps, respectively, and 1.2% (1.0–1.4) and 2.0% (1.6–2.4) among men with no polyps and low-risk polyps, respectively (Table 2). All-site CRC cumulative incidence curves followed a similar pattern to the proximal cancer cumulative incidence curves (Figure 3). Estimates of cumulative all-site CRC incidence at 10 and 20 years after baseline are presented in Table 2.

Compared to women with no polyps, women with low-risk polyps had a higher distal cancer risk (multivariable HR: 1.85 [95%CI 1.18–2.89]), whereas there was no statistically significant difference in distal cancer risk between the two polyp groups in men (multivariable HR: 1.27 [0.87–1.83]). Risk of proximal cancer was higher among women and men with low-risk polyps, compared to those with no polyps (multivariable HRs: 2.08 [1.61–2.68] for women; 1.62 [1.25–2.11] for men) (Table 3). There was little variation in the HRs comparing CRC incidence between the two polyp groups by baseline age group or endoscopist ADR ranking (data not shown).

**Table 3.** Comparing distal, proximal, and all-site colorectal cancer incidence without surveillance between the ‘no polyps’ and low-risk groups in women and men (*n* = 37,954).

Subsite	Sex	Polyp group^a^^,^^b^	No. of cases	Incidence rate per 100,000 person-years (95%CI)	Univariable HR (95%CI)	*p*-value^c^	Multivariable HR (95%CI)^d^	*p*-value^c^^,^^d^
Distal	Women	No polyps	81	26 (21–32)	1	0.02	1	0.01
		Low-risk	26	44 (30–65)	1.74 (1.12–2.71)		1.85 (1.18–2.89)	
	Men	No polyps	102	40 (33–49)	1	0.52	1	0.22
		Low-risk	40	45 (33–61)	1.13 (0.78–1.63)		1.27 (0.87–1.83)	
Proximal	Women	No polyps	212	68 (59–77)	1	<0.001	1	<0.001
		Low-risk	82	140 (112–173)	2.10 (1.62–2.70)		2.08 (1.61–2.68)	
	Men	No polyps	154	61 (52–71)	1	<0.001	1	<0.001
		Low-risk	88	98 (80–121)	1.64 (1.26–2.13)		1.62 (1.25–2.11)	
All-site	Women	No polyps	302	96 (86–108)	1	<0.001	1	<0.001
		Low-risk	114	194 (162–233)	2.05 (1.65–2.54)		2.03 (1.62–2.54)	
	Men	No polyps	267	105 (93–119)	1	0.002	1	<0.001
		Low-risk	130	145 (122–172)	1.40 (1.14–1.73)		1.46 (1.18–1.82)	

CI: confidence interval; HR: hazard ratio; CRC: colorectal cancer; ADR: adenoma detection rate.

^a^ Participants in the low-risk group had polyps detected at baseline that did not meet the following high-risk criteria: an adenoma or HP ≥10 mm, ≥3 adenomas, or an adenoma with villous/tubulovillous histology or high-grade dysplasia. Participants in the ‘no polyps’ group had no polyps detected at baseline.

^b^ Each participant's follow-up time was included from start of time-at-risk and censored at any first surveillance visit; in total, there were 313,279, 253,445, 58,710, and 89,543 person-years in the analyses including women with no polyps, men with no polyps, women with low-risk polyps, and men with low-risk polyps, respectively.

^c^ Calculated with the likelihood ratio test.

^d^ The multivariable HRs were from multivariable models, run separately in women and men, adjusted for baseline age group and any baseline patient or procedural characteristics independently associated with distal, proximal, or all-site CRC incidence among all participants in the two polyp groups combined. For distal cancer incidence, the final multivariable model contained polyp group, age group, family history of CRC, and endoscopist ADR ranking group; for proximal cancer incidence, the final multivariable model contained polyp group, age group, and family history of CRC; and for all-site CRC incidence, the final multivariable model contained polyp group, age group, family history of CRC, endoscopist ADR ranking group, bowel preparation quality, and duration of the baseline visit. The associated *p*-values were for the inclusion of the ‘polyp group’ variable in the models. We identified the characteristics that were independently associated with distal, proximal, or all-site CRC incidence through backward selection from preliminary models including all participants in the two polyp groups combined, retaining variables with *p*-values ≤0.05 from likelihood ratio tests (data not shown). The proportionality assumption was violated for two baseline characteristics (family history of CRC and bowel preparation quality) for the outcome of distal cancer incidence and for one baseline characteristic (family history of CRC) for the outcome of all-site CRC incidence in univariable models; however, based on visual inspection of the Kaplan–Meier plots for these variables (data not shown), they were assessed to not be significant violations and no adjustments were made.

## Discussion

This analysis included ∼39,000 participants who had no polyps (76%), low-risk polyps (21%), or high-risk polyps (4%) in the distal colorectum at FS screening and were followed up for 21 years, mostly (97%) without colonoscopy surveillance. It provides unique data on long-term post-screening CRC risk, largely unperturbed by surveillance, and by polyp group, anatomic subsite, and sex.

Compared to the general population, distal cancer risk was substantially lower (by ∼50–75%) among women and men with either no distal polyps or low-risk distal polyps at screening, without any surveillance. This, together with the overall UKFSST findings which show that screening with polypectomy substantially reduced distal cancer risk,^14,16,17^ supports the effectiveness of endoscopic screening, with polypectomy when needed, in providing sustained protection against cancer development in the examined regions of the colorectum.

Compared to men with no distal polyps, men with low-risk distal polyps had a similar risk of distal cancer. In contrast, women with low-risk distal polyps had a ∼2-fold higher distal cancer risk than those without distal polyps, with the cumulative incidence curve for women with low-risk polyps diverging from the curve for women with no polyps ∼15 years after baseline. This might have contributed to a pattern observed in the overall 21-year UKFSST findings; namely, the weakening in effect of FS on distal cancer incidence seen after ∼15 years.^17^ This was mostly due to a weakening in effect in women, which might, in turn, have particularly occurred among women with low-risk distal polyps. These findings suggest that among those with low-risk distal polyps, men had developed most of their polyps by the time of screening, whereas women were more likely to continue developing polyps post-screening.

Among women and men without distal polyps, proximal cancer risk was no higher compared to that in the general population. Among men with low-risk distal polyps, proximal cancer risk was not significantly different from that in the general population, although the SIR point estimate and 95%CI suggested that proximal cancer risk might have been higher than in the general population. However, this should be interpreted with caution because the difference was not statistically significant. Among women with low-risk distal polyps, proximal cancer risk was ∼2-fold higher than in the general population and this was consistent across baseline characteristics. These unique data suggest that detection of low-risk distal polyps at screening in women indicates increased proximal cancer risk; in such cases, a colonoscopy may provide protection against proximal cancer.

If women with low-risk distal polyps had developed most of their proximal polyps by the time of screening, an index colonoscopy, rather than surveillance colonoscopy, may have been more optimal. This is plausible because proximal cancer cumulative incidence curves for the two polyp groups in women began diverging ∼5 years after baseline, within estimated polyp-to-cancer transition times.^23^ Distal cancer cumulative incidence curves for the polyp groups in women started diverging ∼10 years later. It could be that among women with low-risk distal polyps, de novo neoplastic development post-screening made a greater contribution to distal cancer risk, with more of the proximal cancer risk deriving from polyps present at screening.

Had women with low-risk distal polyps been referred for index colonoscopy in the UKFSST, it could have required 3121 additional colonoscopies, nearly tripling the total number of index colonoscopies. The additional resources, costs, and risks of colonoscopy-associated complications would need to be weighed against possible clinical benefits. In another FS trial in which everyone with polyps was referred for colonoscopy, borderline statistically significant reductions in proximal cancer incidence were observed among invited-to-screening participants, compared with controls, after 16 years; however, there was substantial endoscopy use among controls.^24^

Women and men with low-risk distal polyps had a ∼2-fold higher proximal cancer risk than those without distal polyps, indicating they were more prone to synchronous and/or metachronous proximal polyps, which, left intact, likely contributed to the greater differences in risk between the two polyp groups seen for proximal than distal cancer. The particularly high proximal cancer risk in women with low-risk distal polyps could reflect multiple factors. Proximal cancers account for a greater proportion of CRCs in women than in men, seen in the present analysis and existing literature.^25^ Associations of age, oestrogen status, and reproductive history with proximal cancer risk among women have led to suggestions that oestrogens might be involved.^25–27^ Dietary and lifestyle factors might exert differential effects on CRC risk according to sex and/or subsite.^28^ Women are more prone to CRCs with high microsatellite instability, which predominantly occur in the proximal colon, with suggestions that oestrogens might be involved.^25,28^ Data were unavailable for this analysis to assess these contributions to CRC risk.

Proximal cancer risk appeared to be higher among participants with small (5–9 mm) distal adenomas or HPs than those with diminutive (<5 mm) distal adenomas or HPs. A possible hypothesis is that small distal polyps were more strongly associated with the presence of synchronous and/or metachronous proximal polyps than diminutive distal polyps. However, this should be interpreted with caution because these observations could reflect multiple testing. The UK, US, and European surveillance guidelines consider diminutive and small polyps to carry equivalent CRC risk but highlight the uncertainty due to insufficient data.^2–4^

Studies examining post-polypectomy CRC outcomes usually report people with low-risk polyps (1–2 adenomas <10 mm, tubular histology, low-grade dysplasia) to have a similar or lower CRC risk compared to the general population or people without polyps.^7,11–13,29–33^ Limited sample sizes and follow-up periods often meant numbers of CRC cases were insufficient for precise estimates or subgroup analyses. Post-polypectomy surveillance was frequently performed in these studies, which likely lowered CRC risk, but this was often not addressed in analyses. Therefore, as suggested in US surveillance guidelines, the low CRC risk among people with low-risk polyps might partly be due to exposure to surveillance.^4^ A 2021 study examining post-polypectomy CRC risk, censoring at adenoma removal during follow-up, reported findings more similar to ours; namely, that women with low-risk adenomas had a higher CRC risk, and equivalent men a lower risk, compared to the general population. However, most participants had a baseline colonoscopy for symptom investigation and subsite-specific analyses were not performed.^34^

In the high-risk group, all-site CRC risk was not statistically significantly different from that in the general population. This was true when including all high-risk participants and when restricting to those who attended surveillance or those who did not attend surveillance. However, for all high-risk participants and those who attended surveillance, SIR point estimates and 95%CIs suggested that all-site CRC risk might have been lower than in the general population. The results from analyses of high-risk participants who did not attend surveillance should be interpreted with caution because there were limited person-years and CRC cases, resulting in imprecise estimates of CRC risk.

Our study has limitations. We excluded individuals with inflammatory bowel disease, polyposis, or Lynch syndrome based on endoscopist comments; we might have missed individuals if their condition was not reported. Although screening examinations were performed in the 1990s, 87% of participants had ≥1 complete screening examination with at least adequate bowel preparation. Surveillance data were captured through 2012 so we might be missing examinations; however, it is unlikely many had surveillance post-2012 because they would be aged 68–79 years and surveillance is recommended to stop around 75 years.^2^ We might be missing data if participants had surveillance at hospitals outside the trial centres. Unavailability of comorbidity and dietary and lifestyle data prevented assessment of their contributions to CRC risk. Limited awareness of serrated polyps in the trial era prevented understanding of the associated CRC risks.

This analysis included ∼39,000 participants from 14 UK centres, 21 years’ follow-up, and 870 incident CRCs. This enabled various subgroup analyses, revealing unique insights into long-term CRC risk by polyp group, subsite, and sex. We previously found that among UKFSST participants recruited in England, <2% had ever tested positive with the guaiac faecal occult blood test (gFOBT) within the BCSP through 2015, and only 4% were within the eligible age range for screening beyond 2015.^17^ Therefore, the proportion of participants in the present analysis who had colonoscopy performed through the BCSP for positive gFOBTs is low. As only 0.3% of the ‘no polyps’/low-risk groups underwent surveillance, this enabled examination of CRC risk unperturbed by surveillance, which was of great value in understanding whether screening examinations alone were sufficient to protect against CRC.

## Conclusions

Over 21 years after FS screening, all-site CRC risk was not statistically significantly different among participants who had high-risk polyps at screening, compared to that in the general population, regardless of whether surveillance was accounted for. However, analyses of high-risk participants who did not attend surveillance were limited by the low count of person-years and CRC cases. Among women and men with either no distal polyps or low-risk distal polyps at screening, distal cancer risk was low, without surveillance, compared to the general population. While proximal cancer risk among women and men with no distal polyps, and among men with low-risk distal polyps, was either lower or not statistically significantly different compared to that in the general population, it was ∼2-fold higher among women with low-risk distal polyps. These women may have benefitted from having a colonoscopy.

## Supplemental Material

sj-docx-1-msc-10.1177_09691413251316442 - Supplemental material for Long-term colorectal cancer incidence in a post-endoscopic screening cohort, accounting for surveillance, by baseline polyp group, anatomic subsite, and sex Supplemental material, sj-docx-1-msc-10.1177_09691413251316442 for Long-term colorectal cancer incidence in a post-endoscopic screening cohort, accounting for surveillance, by baseline polyp group, anatomic subsite, and sex by Emma C Robbins, Kate Wooldrage, Brian P Saunders and Amanda J Cross in Journal of Medical Screening
