# Supplementary material for: Long-term colorectal cancer incidence in a post-endoscopic screening cohort, accounting for surveillance, by baseline polyp group, anatomic subsite, and sex
Source: J Med Screen. 2025 Jan 28;32(3):150–60. doi: 10.1177/09691413251316442 (PMC12354984; doi:10.1177/09691413251316442)
Supplement: sj-docx-1-msc-10.1177_09691413251316442 - Supplemental material for Long-term colorectal cancer incidence in a post-endoscopic screening cohort, accounting for surveillance, by baseline polyp group, anatomic subsite, and sex [file sj-docx-1-msc-10.1177_09691413251316442.docx]

**Supplementary Table 1.** **Baseline patient, procedural, and polyp characteristics in the ‘no polyps’, low-risk, and high-risk groups by sex (n=39,417)**

|  | **‘No polyps’^a^** | | **Low-risk^a^** | | **High-risk^a^** | |
| --- | --- | --- | --- | --- | --- | --- |
| **Baseline characteristic** | **Women** | **Men** | **Women** | **Men** | **Women** | **Men** |
| Total | 16,059 (100.0) | 13,733 (100.0) | 3,121 (100.0) | 5,041 (100.0) | 449 (100.0) | 1,014 (100.0) |
| Age group at baseline, years |  |  |  |  |  |  |
| 55-59 | 7,402 (46.1) | 6,468 (47.1) | 1,379 (44.2) | 2,274 (45.1) | 204 (45.4) | 413 (40.7) |
| 60-66 | 8,657 (53.9) | 7,265 (52.9) | 1,742 (55.8) | 2,767 (54.9) | 245 (54.6) | 601 (59.3) |
| Family history of CRC^b^ |  |  |  |  |  |  |
| No | 13,233 (82.4) | 11,545 (84.1) | 2,522 (80.8) | 4,151 (82.3) | 366 (81.5) | 813 (80.2) |
| Yes | 1,782 (11.1) | 1,338 (9.7) | 389 (12.5) | 580 (11.5) | 53 (11.8) | 133 (13.1) |
| Missing | 1,044 (6.5) | 850 (6.2) | 210 (6.7) | 310 (6.1) | 30 (6.7) | 68 (6.7) |
| Endoscopist ADR ranking group^c^ |  |  |  |  |  |  |
| High | 5,484 (34.1) | 4,486 (32.7) | 1,513 (48.5) | 2,307 (45.8) | 201 (44.8) | 447 (44.1) |
| Intermediate | 4,827 (30.1) | 4,145 (30.2) | 885 (28.4) | 1,534 (30.4) | 137 (30.5) | 254 (25.0) |
| Low | 5,143 (32.0) | 4,628 (33.7) | 628 (20.1) | 1,080 (21.4) | 95 (21.2) | 280 (27.6) |
| Missing | 605 (3.8) | 474 (3.5) | 95 (3.0) | 120 (2.4) | 16 (3.6) | 33 (3.3) |
| Index colonoscopy |  |  |  |  |  |  |
| No | 16,032 (99.8) | 13,713 (99.9) | 3,058 (98.0) | 4,918 (97.6) | 0 (0.0) | 0 (0.0) |
| Yes | 27 (0.2) | 20 (0.1) | 63 (2.0) | 123 (2.4) | 449 (100.0) | 1,014 (100.0) |
| No. of examinations during baseline visit |  |  |  |  |  |  |
| 1 | 15,359 (95.6) | 13,085 (95.3) | 2,821 (90.4) | 4,487 (89.0) | 0 (0.0) | 0 (0.0) |
| 2 | 671 (4.2) | 619 (4.5) | 275 (8.8) | 497 (9.9) | 361 (80.4) | 814 (80.3) |
| ≥3 | 29 (0.2) | 29 (0.2) | 25 (0.8) | 57 (1.1) | 88 (19.6) | 200 (19.7) |
| Duration of baseline visit, days |  |  |  |  |  |  |
| 1 | 15,802 (98.4) | 13,479 (98.2) | 2,934 (94.0) | 4,711 (93.5) | 0 (0.0) | 0 (0.0) |
| 2 – 90 | 236 (1.5) | 230 (1.7) | 149 (4.8) | 264 (5.2) | 324 (72.2) | 707 (69.7) |
| 91 – 334 | 21 (0.1) | 24 (0.2) | 38 (1.2) | 66 (1.3) | 125 (27.8) | 307 (30.3) |
| Bowel preparation quality^d^ |  |  |  |  |  |  |
| Excellent | 7,194 (44.8) | 5,573 (40.6) | 1,216 (39.0) | 1,885 (37.4) | 126 (28.1) | 226 (22.3) |
| Good | 5,367 (33.4) | 4,679 (34.1) | 1,145 (36.7) | 1,846 (36.6) | 194 (43.2) | 473 (46.6) |
| Adequate | 2,954 (18.4) | 3,108 (22.6) | 577 (18.5) | 1,033 (20.5) | 84 (18.7) | 234 (23.1) |
| Poor | 419 (2.6) | 253 (1.8) | 30 (1.0) | 39 (0.8) | 15 (3.3) | 39 (3.8) |
| Missing | 125 (0.8) | 120 (0.9) | 153 (4.9) | 238 (4.7) | 30 (6.7) | 42 (4.1) |
| Examination completeness^d^ |  |  |  |  |  |  |
| Complete | 13,447 (83.7) | 12,781 (93.1) | 2,671 (85.6) | 4,797 (95.2) | 397 (88.4) | 938 (92.5) |
| Incomplete | 2,568 (16.0) | 908 (6.6) | 426 (13.6) | 193 (3.8) | 47 (10.5) | 70 (6.9) |
| Missing | 44 (0.3) | 44 (0.3) | 24 (0.8) | 51 (1.0) | 5 (1.1) | 6 (0.6) |
| No. of adenomas |  |  |  |  |  |  |
| 0 | 16,059 (100.0) | 13,733 (100.0) | 2,039 (65.3)^e^ | 3,025 (60.0)^e^ | 14 (3.1) | 30 (3.0) |
| 1 | 0 (0.0) | 0 (0.0) | 995 (31.9) | 1,751 (34.7) | 302 (67.3) | 484 (47.7) |
| 2 | 0 (0.0) | 0 (0.0) | 87 (2.8) | 265 (5.3) | 88 (19.6) | 238 (23.5) |
| 3-4 | 0 (0.0) | 0 (0.0) | 0 (0.0) | 0 (0.0) | 39 (8.7) | 197 (19.4) |
| 5-9 | 0 (0.0) | 0 (0.0) | 0 (0.0) | 0 (0.0) | 6 (1.3) | 65 (6.4) |
| Adenoma size, mm^f^ |  |  |  |  |  |  |
| <5 | “” | “” | 667 (61.6) | 1,170 (58.0) | 42 (9.7) | 78 (7.9) |
| 5-9 | “” | “” | 415 (38.4) | 846 (42.0) | 106 (24.4) | 211 (21.4) |
| ≥10 | “” | “” | “” | “” | 287 (66.0) | 695 (70.6) |
| No. of HPs |  |  |  |  |  |  |
| 0 | 16,059 (100.0) | 13,733 (100.0) | 1,003 (32.1) | 1,578 (31.3) | 330 (73.5) | 628 (61.9) |
| 1-2 | 0 (0.0) | 0 (0.0) | 1,780 (57.0) | 2,753 (54.6) | 82 (18.3) | 253 (25.0) |
| 3-19 | 0 (0.0) | 0 (0.0) | 338 (10.8) | 710 (14.1) | 37 (8.2) | 133 (13.1) |
| HP size, mm^f^ |  |  |  |  |  |  |
| <5 | “” | “” | 1,748 (82.5) | 2,690 (77.7) | 64 (53.8) | 194 (50.3) |
| 5-9 | “” | “” | 370 (17.5) | 773 (22.3) | 28 (23.5) | 119 (30.8) |
| ≥10 | “” | “” | “” | “” | 27 (22.7) | 73 (18.9) |
| Location of HPs^g^ |  |  |  |  |  |  |
| RM/RS only | “” | “” | 1,130 (53.4) | 1,643 (47.4) | 43 (36.1) | 122 (31.6) |
| Not confined to RM/RS | “” | “” | 988 (46.6) | 1,820 (52.6) | 76 (63.9) | 264 (68.4) |
| Presence of adenomas and/or HPs |  |  |  |  |  |  |
| No adenomas or HPs | 16,059 (100.0) | 13,733 (100.0) | 117 (3.7)^h^ | 145 (2.9)^h^ | 0 (0.0) | 0 (0.0) |
| Adenomas only | 0 (0.0) | 0 (0.0) | 886 (28.4) | 1,433 (28.4) | 330 (73.5) | 628 (61.9) |
| HPs only | 0 (0.0) | 0 (0.0) | 1,922 (61.6) | 2,880 (57.1) | 14 (3.1) | 30 (3.0) |
| Adenomas and HPs | 0 (0.0) | 0 (0.0) | 196 (6.3) | 583 (11.6) | 105 (23.4) | 356 (35.1) |

ADR: adenoma detection rate. CRC: colorectal cancer. HP: hyperplastic polyp. mm: millimetre. RM: rectum. RS: rectosigmoid.

Comparing patient, procedural, and polyp characteristics between women and men within the three polyp groups, using the χ^2^ test, the following comparisons had p-values ≤0.05: family history of CRC, endoscopist ADR ranking group, bowel preparation quality, and examination completeness in the ‘no polyps’ group; endoscopist ADR ranking group, examination completeness, number of adenomas, adenoma size, number of HPs, HP size, HP location, and presence of adenomas and/or HPs in the low-risk group; and endoscopist ADR ranking group, bowel preparation quality, examination completeness, number of adenomas, number of HPs, and presence of adenomas and/or HPs in the high-risk group. Comparing patient and procedural characteristics between the three polyp groups, within women and within men, using the χ^2^ test, all comparisons had p-values ≤0.05, except for the comparisons for baseline age group in women and family history of CRC in women.

^a^Participants in the high-risk group had an adenoma or HP ≥10mm, ≥3 adenomas, or an adenoma with villous/tubulovillous histology or high-grade dysplasia at baseline. Participants in the low-risk group had polyps detected at baseline that did not meet the high-risk criteria. Participants in the ‘no polyps’ group had no polyps detected at baseline.

^b^Defined as a history of CRC in a first-degree relative, as reported by participants on a pre-screening questionnaire.

^c^Endoscopists were classified into high-, intermediate-, or low-detector ranking groups based on their ADR (with ADR calculated as the % of participants screened by the endoscopist who had ≥1 adenomas detected during screening).

^d^For participants in the ‘no polyps’ and low-risk groups who had flexible sigmoidoscopy (FS) at baseline and no index colonoscopy, bowel preparation quality and examination completeness were defined, respectively, according to the best quality preparation and most complete examination for an FS performed during the baseline visit. For those in the ‘no polyps’ and low-risk groups who had FS and an index colonoscopy at baseline, bowel preparation quality and examination completeness were defined, respectively, according to the best quality preparation and most complete examination for an FS or colonoscopy performed during the baseline visit. For the high-risk group, these characteristics were defined according to the best quality preparation and most complete examination for a colonoscopy performed during the baseline visit.

^e^Of women and men in the low-risk group with no adenomas, 94% (1,922/2,039) and 95% (2,880/3,025) had HPs, respectively.

^f^Defined according to the largest diameter recorded for an adenoma or HP during the baseline visit; percentages were calculated using the number of participants with at least one baseline adenoma or HP as the denominator.

^g^Percentages were calculated using the number of participants with at least one baseline HP as the denominator.

^h^Of participants in the low-risk group who had no adenomas or HPs, the majority had a baseline polyp reported to be an ‘assume polyp’; most were diagnosed as adenomas at endoscopy sighting but were lost after removal, preventing pathological confirmation of polyp type.

**Supplementary Table 2.** **Baseline patient, procedural, and polyp characteristics in the high-risk group by attendance at surveillance (n=1,463)^a^**

|  | **Attended ≥1 surveillance visits**  n (%) | **Attended no surveillance visits**  n (%) | **p-value^b^** |
| --- | --- | --- | --- |
|  |  |  |  |
| Total | 1,190 (100.0) | 273 (100.0) |  |
| Age group at baseline, years |  |  | 0.07 |
| 55-59 | 515 (43.3) | 102 (37.4) |  |
| 60-66 | 675 (56.7) | 171 (62.6) |  |
| Sex |  |  | 0.64 |
| Women | 362 (30.4) | 87 (31.9) |  |
| Men | 828 (69.6) | 186 (68.1) |  |
| Family history of CRC^c^ |  |  | 0.77 |
| No | 961 (80.8) | 218 (79.9) |  |
| Yes | 152 (12.8) | 34 (12.5) |  |
| Missing | 77 (6.5) | 21 (7.7) |  |
| Endoscopist ADR ranking group^d^ |  |  | 0.29 |
| High | 539 (45.3) | 109 (39.9) |  |
| Intermediate | 310 (26.1) | 81 (29.7) |  |
| Low | 299 (25.1) | 76 (27.8) |  |
| Missing | 42 (3.5) | 7 (2.6) |  |
| No. of examinations during baseline visit |  |  | 0.16 |
| 2 | 964 (81.0) | 211 (77.3) |  |
| ≥3 | 226 (19.0) | 62 (22.7) |  |
| Duration of baseline visit, days |  |  | 0.17 |
| 2 – 90 | 848 (71.3) | 183 (67.0) |  |
| 91 – 334 | 342 (28.7) | 90 (33.0) |  |
| Bowel preparation quality^e^ |  |  | 0.94 |
| Excellent | 286 (24.0) | 66 (24.2) |  |
| Good | 546 (45.9) | 121 (44.3) |  |
| Adequate | 259 (21.8) | 59 (21.6) |  |
| Poor | 43 (3.6) | 11 (4.0) |  |
| Missing | 56 (4.7) | 16 (5.9) |  |
| Examination completeness^e^ |  |  | 0.001 |
| Complete | 1,100 (92.4) | 235 (86.1) |  |
| Incomplete or missing^f^ | 90 (7.6) | 38 (13.9) |  |
| No. of adenomas |  |  | 0.03 |
| <3 | 927 (77.9) | 229 (83.9) |  |
| 3-9 | 263 (22.1) | 44 (16.1) |  |
| Adenoma ≥10mm^g^ |  |  | <0.001 |
| No | 360 (30.3) | 121 (44.3) |  |
| Yes | 830 (69.7) | 152 (55.7) |  |
| Adenoma with villous/tubulovillous histology^g^ |  |  | 0.63 |
| No | 517 (43.4) | 123 (45.1) |  |
| Yes | 673 (56.6) | 150 (54.9) |  |
| Adenoma with high-grade dysplasia^g^ |  |  | 0.12 |
| No | 1,011 (85.0) | 242 (88.6) |  |
| Yes | 179 (15.0) | 31 (11.4) |  |
| Proximal adenoma^h^ |  |  | 0.20 |
| No | 953 (80.1) | 228 (83.5) |  |
| Yes | 237 (19.9) | 45 (16.5) |  |
| No. of HPs |  |  | 0.005 |
| <3 | 1,065 (89.5) | 228 (83.5) |  |
| 3-19 | 125 (10.5) | 45 (16.5) |  |
| HP ≥10mm^i^ |  |  | <0.001 |
| No | 1,131 (95.0) | 232 (85.0) |  |
| Yes | 59 (5.0) | 41 (15.0) |  |
| Location of HPs^j^ |  |  | 0.08 |
| RM/RS only | 137 (34.6) | 28 (25.7) |  |
| Not confined to RM/RS | 259 (65.4) | 81 (74.3) |  |
| Presence of adenomas and/or HPs |  |  | <0.001 |
| No adenomas or HPs | 0 (0.0) | 0 (0.0) |  |
| Adenomas only | 794 (66.7) | 164 (60.1) |  |
| HPs only | 18 (1.5) | 26 (9.5) |  |
| Adenomas and HPs | 378 (31.8) | 83 (30.4) |  |

ADR: adenoma detection rate. CRC: colorectal cancer. HP: hyperplastic polyp. mm: millimetre. RM: rectum. RS: rectosigmoid.

^a^Participants in the high-risk group had an adenoma or HP ≥10mm, ≥3 adenomas, or an adenoma with villous/tubulovillous histology or high-grade dysplasia at baseline.

^b^Calculated with the χ^2^ test.

^c^Defined as a history of CRC in a first-degree relative, as reported by participants on a pre-screening questionnaire.

^d^Endoscopists were classified into high-, intermediate-, or low-detector ranking groups based on their ADR (with ADR calculated as the % of participants screened by the endoscopist who had ≥1 adenomas detected during screening).

^e^Bowel preparation quality and examination completeness were defined, respectively, according to the best quality preparation and most complete examination for a colonoscopy performed during the baseline visit.

^f^The ‘incomplete’ and ‘missing’ categories were grouped due to low cell counts.

^g^Adenoma size, histology, and dysplasia were defined, respectively, according to the largest diameter, greatest degree of villous architecture, and highest grade of dysplasia recorded during the baseline visit.

^h^Any adenoma found at a site proximal to the sigmoid colon at index colonoscopy.

^i^Defined according to the largest diameter recorded for a HP during the baseline visit.

^j^Percentages were calculated using the number of participants with at least one baseline HP as the denominator.

**Supplementary Table 3. Characteristics of all CRCs diagnosed among women and men in the ‘no polyps’, low-risk, and high-risk groups including all follow-up time (n=39,417)**

| **Characteristic of CRC** | **Women** | | | **Men** | | |
| --- | --- | --- | --- | --- | --- | --- |
|  | **No polyps^a^**  n (%) | **Low-risk^a^**  n (%) | **High-risk^a^**  n (%) | **No polyps^a^**  n (%) | **Low-risk^a^**  n (%) | **High-risk^a^**  n (%) |
| Overall total | 302 (100.0) | 116^b^ (100.0) | 9 (100.0) | 269^c^ (100.0) | 133^d^ (100.0) | 41 (100.0) |
| Anatomic site |  |  |  |  |  |  |
| Distal | 81 (26.8) | 27^b^ (22.8) | 2 (27.3) | 102 (38.1) | 42^d^ (31.6) | 13 (31.7) |
| Proximal | 212 (70.2) | 83^b^ (71.9) | 6 (63.6) | 154 (57.0) | 88 (66.2) | 26 (63.4) |
| Colon, unspecified | 9 (3.0) | 6 (5.3) | 1 (9.1) | 13 (4.8) | 3 (2.3) | 2 (4.9) |
| Morphology |  |  |  |  |  |  |
| Adenocarcinoma | 274 (90.7) | 105 (90.5) | 6 (66.7) | 246 (91.4) | 117 (88.0) | 39 (95.1) |
| Cancer, unspecified | 28 (9.3) | 11 (9.5) | 3 (33.3) | 23 (8.6) | 16 (12.0) | 2 (4.9) |

CRC: colorectal cancer.

^a^Participants in the high-risk group had an adenoma or HP ≥10mm, ≥3 adenomas, or an adenoma with villous/tubulovillous histology or high-grade dysplasia at baseline. Participants in the low-risk group had polyps detected at baseline that did not meet the high-risk criteria. Participants in the ‘no polyps’ group had no polyps detected at baseline.

^b^This table includes one extra distal cancer, one extra proximal cancer, and two extra all-site cancers among women in the low-risk group that are not included in Tables 2-3, Figure 3, or Supplementary Tables 4-5 because one woman in this group was diagnosed with distal cancer after attending surveillance, while another woman in this group was diagnosed with proximal cancer after attending surveillance, and these cancers were not included in analyses of CRC incidence without surveillance.

^c^This table includes two extra all-site cancers among men in the ‘no polyps’ group that are not included in Tables 2-3, Figure 3, or Supplementary Tables 4-5 because two men in this group were diagnosed with synchronous distal and proximal cancers which were all counted in the present table, although each of these men counted as one all-site cancer case in analyses of CRC incidence.

^d^This table includes two extra distal cancers and three extra all-site cancers among men in the low-risk group that are not included in Tables 2-3, Figure 3, or Supplementary Tables 4-5 because two men in this group were diagnosed with distal cancer after attending surveillance, while another man in this group was diagnosed with synchronous distal and proximal cancers.

**Supplementary Table 4.** **Distal cancer incidence without surveillance and age-sex-standardised incidence ratios in women and men in the ‘no polyps’ and low-risk groups, by baseline patient, procedural, and polyp characteristics (n=37,954)**

| **Polyp group^a^** | **Sex** | **Baseline characteristic** | **n (%)** | **No. of person-years^b^** | **Total no. of cases** | **Incidence rate per 100,000 person-years (95%CI)** | **Follow-up** | |  | | **Standardisation** | |
| --- | --- | --- | --- | --- | --- | --- | --- | --- | --- | --- | --- | --- |
|  |  |  |  |  |  |  | 10 years | | 20 years | |  |  |
|  |  |  |  |  |  |  | No. of cases | Cumulative incidence, % (95%CI) | No. of cases | Cumulative incidence, % (95%CI) | No. of expected cases | SIR (95%CI) |
| No polyps | Women | Total | 16,059 (100.0) | 313,279 | 81 | 26 (21-32) | 20 | 0.1 (0.1-0.2) | 75 | 0.5 (0.4-0.7) | 270 | 0.30 (0.24-0.37) |
|  |  | Age group at baseline, years |  |  |  |  |  |  |  |  |  |  |
|  |  | 55-59 | 7,402 (46.1) | 147,762 | 33 | 22 (16-31) | 6 | 0.1 (0.0-0.2) | 28 | 0.4 (0.3-0.6) | 112 | 0.29 (0.20-0.41) |
|  |  | 60-66 | 8,657 (53.9) | 165,517 | 48 | 29 (22-38) | 14 | 0.2 (0.1-0.3) | 47 | 0.6 (0.5-0.8) | 158 | 0.30 (0.22-0.40) |
|  |  | Endoscopist ADR ranking group^c,d^ |  |  |  |  |  |  |  |  |  |  |
|  |  | High | 5,484 (34.1) | 107,155 | 21 | 20 (13-30) | 4 | 0.1 (0.0-0.2) | 20 | 0.4 (0.3-0.6) | 93 | 0.23 (0.14-0.35) |
|  |  | Intermediate | 4,827 (30.1) | 94,721 | 23 | 24 (16-37) | 5 | 0.1 (0.0-0.3) | 21 | 0.5 (0.3-0.8) | 82 | 0.28 (0.18-0.42) |
|  |  | Low | 5,143 (32.0) | 99,240 | 34 | 34 (24-48) | 9 | 0.2 (0.1-0.4) | 31 | 0.7 (0.5-1.0) | 85 | 0.40 (0.28-0.56) |
|  |  | Bowel preparation quality^c,e^ |  |  |  |  |  |  |  |  |  |  |
|  |  | Excellent | 7,194 (44.8) | 141,930 | 35 | 25 (18-34) | 5 | 0.1 (0.0-0.2) | 32 | 0.5 (0.4-0.7) | 122 | 0.29 (0.20-0.40) |
|  |  | Good | 5,367 (33.4) | 104,279 | 29 | 28 (19-40) | 7 | 0.1 (0.1-0.3) | 26 | 0.6 (0.4-0.8) | 90 | 0.32 (0.22-0.46) |
|  |  | Adequate | 2,954 (18.4) | 56,778 | 13 | 23 (13-39) | 6 | 0.2 (0.1-0.5) | 13 | 0.5 (0.3-0.8) | 49 | 0.27 (0.14-0.45) |
|  |  | Poor | 419 (2.6) | 7,814 | 2 | 26 (6-102) | 1 | 0.3 (0.0-1.8) | 2 | 0.6 (0.1-2.3) | 7 | 0.30 (0.04-1.08) |
|  |  | Examination completeness^c,e^ |  |  |  |  |  |  |  |  |  |  |
|  |  | Complete | 13,447 (83.7) | 263,653 | 64 | 24 (19-31) | 14 | 0.1 (0.1-0.2) | 60 | 0.5 (0.4-0.7) | 227 | 0.28 (0.22-0.36) |
|  |  | Incomplete | 2,568 (16.0) | 48,785 | 16 | 33 (20-54) | 6 | 0.3 (0.1-0.6) | 14 | 0.6 (0.4-1.1) | 42 | 0.38 (0.22-0.62) |
|  | Men | Total | 13,733 (100.0) | 253,445 | 102 | 40 (33-49) | 28 | 0.2 (0.2-0.3) | 86 | 0.7 (0.6-0.9) | 423 | 0.24 (0.20-0.29) |
|  |  | Age group at baseline, years |  |  |  |  |  |  |  |  |  |  |
|  |  | 55-59 | 6,468 (47.1) | 124,401 | 42 | 34 (25-46) | 12 | 0.2 (0.1-0.3) | 35 | 0.6 (0.4-0.8) | 183 | 0.23 (0.17-0.31) |
|  |  | 60-66 | 7,265 (52.9) | 129,043 | 60 | 46 (36-60) | 16 | 0.2 (0.1-0.4) | 51 | 0.9 (0.7-1.2) | 240 | 0.25 (0.19-0.32) |
|  |  | Endoscopist ADR ranking group^c,d^ |  |  |  |  |  |  |  |  |  |  |
|  |  | High | 4,486 (32.7) | 82,774 | 23 | 28 (18-42) | 7 | 0.2 (0.1-0.3) | 21 | 0.5 (0.4-0.8) | 138 | 0.17 (0.11-0.25) |
|  |  | Intermediate | 4,145 (30.2) | 78,110 | 30 | 38 (27-55) | 6 | 0.2 (0.1-0.3) | 23 | 0.7 (0.4-1.0) | 131 | 0.23 (0.15-0.33) |
|  |  | Low | 4,628 (33.7) | 83,621 | 48 | 57 (43-76) | 15 | 0.4 (0.2-0.6) | 41 | 1.1 (0.8-1.5) | 138 | 0.35 (0.26-0.46) |
|  |  | Bowel preparation quality^c,e^ |  |  |  |  |  |  |  |  |  |  |
|  |  | Excellent | 5,573 (40.6) | 104,926 | 38 | 36 (26-50) | 10 | 0.2 (0.1-0.4) | 31 | 0.7 (0.5-0.9) | 175 | 0.22 (0.15-0.30) |
|  |  | Good | 4,679 (34.1) | 85,661 | 40 | 47 (34-64) | 7 | 0.2 (0.1-0.3) | 35 | 0.9 (0.7-1.3) | 142 | 0.28 (0.20-0.38) |
|  |  | Adequate | 3,108 (22.6) | 56,269 | 20 | 36 (23-55) | 9 | 0.3 (0.2-0.6) | 16 | 0.6 (0.4-1.0) | 95 | 0.21 (0.13-0.33) |
|  |  | Poor | 253 (1.8) | 4,367 | 3 | 69 (22-213) | 2 | 0.9 (0.2-3.4) | 3 | 1.4 (0.4-4.2) | 7 | 0.41 (0.08-1.19) |
|  |  | Examination completeness^c,e^ |  |  |  |  |  |  |  |  |  |  |
|  |  | Complete | 12,781 (93.1) | 236,749 | 92 | 39 (32-48) | 24 | 0.2 (0.1-0.3) | 78 | 0.7 (0.6-0.9) | 395 | 0.23 (0.19-0.29) |
|  |  | Incomplete | 908 (6.6) | 15,923 | 10 | 63 (34-117) | 4 | 0.5 (0.2-1.3) | 8 | 1.0 (0.5-2.1) | 27 | 0.37 (0.18-0.68) |
| Low-risk | Women | Total | 3,121 (100.0) | 58,710 | 26 | 44 (30-65) | 7 | 0.2 (0.1-0.5) | 23 | 0.9 (0.6-1.3) | 50 | 0.52 (0.34-0.76) |
|  |  | Age group at baseline, years |  |  |  |  |  |  |  |  |  |  |
|  |  | 55-59 | 1,379 (44.2) | 26,678 | 10 | 37 (20-70) | 3 | 0.2 (0.1-0.7) | 10 | 0.8 (0.5-1.5) | 20 | 0.50 (0.24-0.92) |
|  |  | 60-66 | 1,742 (55.8) | 32,033 | 16 | 50 (31-82) | 4 | 0.2 (0.1-0.7) | 13 | 0.9 (0.5-1.6) | 30 | 0.53 (0.30-0.86) |
|  |  | Endoscopist ADR ranking group^c,d^ |  |  |  |  |  |  |  |  |  |  |
|  |  | High | 1,513 (48.5) | 28,148 | 7 | 25 (12-52) | 2 | 0.1 (0.0-0.6) | 6 | 0.5 (0.2-1.0) | 24 | 0.29 (0.12-0.60) |
|  |  | Intermediate | 885 (28.4) | 16,967 | 10 | 59 (32-110) | 3 | 0.4 (0.1-1.1) | 9 | 1.2 (0.6-2.3) | 15 | 0.68 (0.33-1.25) |
|  |  | Low | 628 (20.1) | 11,891 | 7 | 59 (28-123) | 2 | 0.3 (0.1-1.3) | 6 | 1.2 (0.5-2.6) | 10 | 0.69 (0.28-1.42) |
|  |  | Bowel preparation quality^c,e^ |  |  |  |  |  |  |  |  |  |  |
|  |  | Excellent | 1,216 (39.0) | 23,292 | 11 | 47 (26-85) | 3 | 0.3 (0.1-0.8) | 9 | 0.9 (0.5-1.7) | 20 | 0.55 (0.27-0.99) |
|  |  | Good | 1,145 (36.7) | 21,467 | 9 | 42 (22-81) | 0 | “” | 8 | 0.9 (0.4-1.7) | 18 | 0.49 (0.22-0.93) |
|  |  | Adequate | 577 (18.5) | 10,497 | 4 | 38 (14-102) | 3 | 0.6 (0.2-1.7) | 4 | 0.9 (0.3-2.4) | 9 | 0.45 (0.12-1.15) |
|  |  | Poor | 30 (1.0) | 584 | 1 | 171 (24-1,216) | 0 | “” | 1 | 3.9 (0.6-24.3) | 1 | 1.93 (0.05-10.75) |
|  |  | Examination completeness^c,e^ |  |  |  |  |  |  |  |  |  |  |
|  |  | Complete | 2,671 (85.6) | 50,421 | 23 | 46 (30-69) | 7 | 0.3 (0.1-0.6) | 21 | 0.9 (0.6-1.4) | 43 | 0.53 (0.34-0.80) |
|  |  | Incomplete | 426 (13.6) | 7,818 | 3 | 38 (12-119) | 0 | “” | 2 | 0.6 (0.2-2.3) | 7 | 0.45 (0.09-1.30) |
|  |  | Adenoma size, mm^f^ |  |  |  |  |  |  |  |  |  |  |
|  |  | No adenomas | 2,039 (65.3) | 38,387 | 11 | 29 (16-52) | 5 | 0.3 (0.1-0.6) | 10 | 0.6 (0.3-1.1) | 33 | 0.34 (0.17-0.60) |
|  |  | <5 | 667 (21.4) | 12,683 | 9 | 71 (37-136) | 2 | 0.3 (0.1-1.3) | 9 | 1.6 (0.8-3.0) | 11 | 0.83 (0.38-1.57) |
|  |  | 5-9 | 415 (13.3) | 7,640 | 6 | 79 (35-175) | 0 | “” | 4 | 1.3 (0.5-3.4) | 7 | 0.91 (0.33-1.98) |
|  |  | No. of HPs |  |  |  |  |  |  |  |  |  |  |
|  |  | 0 | 1,003 (32.1) | 19,114 | 17 | 89 (55-143) | 3 | 0.3 (0.1-1.0) | 15 | 1.8 (1.1-2.9) | 17 | 1.03 (0.60-1.64) |
|  |  | 1-2 | 1,780 (57.0) | 33,786 | 7 | 21 (10-43) | 3 | 0.2 (0.1-0.6) | 6 | 0.4 (0.2-0.8) | 29 | 0.24 (0.10-0.50) |
|  |  | 3-19 | 338 (10.8) | 5,811 | 2 | 34 (9-138) | 1 | 0.3 (0.1-2.3) | 2 | 0.8 (0.2-3.1) | 5 | 0.41 (0.05-1.48) |
|  |  | HP size, mm^g^ |  |  |  |  |  |  |  |  |  |  |
|  |  | <5 | 1,748 (82.5) | 33,059 | 5 | 15 (6-36) | 1 | 0.1 (0.0-0.4) | 4 | 0.3 (0.1-0.7) | 28 | 0.18 (0.06-0.41) |
|  |  | 5-9 | 370 (17.5) | 6,537 | 4 | 61 (23-163) | 3 | 0.9 (0.3-2.8) | 4 | 1.3 (0.5-3.4) | 5 | 0.73 (0.20-1.87) |
|  |  | Location of HPs^h^ |  |  |  |  |  |  |  |  |  |  |
|  |  | RM/RS only | 1,130 (53.4) | 21,251 | 4 | 19 (7-50) | 1 | 0.1 (0.0-0.7) | 4 | 0.4 (0.2-1.1) | 18 | 0.22 (0.06-0.57) |
|  |  | Not confined to RM/RS | 988 (46.6) | 18,346 | 5 | 27 (11-65) | 3 | 0.3 (0.1-1.0) | 4 | 0.5 (0.2-1.2) | 16 | 0.32 (0.10-0.75) |
|  |  | Presence of adenomas and/or HPs |  |  |  |  |  |  |  |  |  |  |
|  |  | No adenomas or HPs^i^ | 117 (3.7) | 2,196 | 2 | 91 (23-364) | 1 | 0.9 (0.1-6.3) | 2 | 2.0 (0.5-7.9) | 2 | 1.04 (0.13-3.76) |
|  |  | Adenomas only | 886 (28.4) | 16,918 | 15 | 89 (53-147) | 2 | 0.2 (0.1-1.0) | 13 | 1.7 (1.0-3.0) | 15 | 1.02 (0.57-1.69) |
|  |  | HPs only | 1,922 (61.6) | 36,191 | 9 | 25 (13-48) | 4 | 0.2 (0.1-0.6) | 8 | 0.5 (0.2-0.9) | 31 | 0.29 (0.13-0.55) |
|  |  | Adenomas and HPs | 196 (6.3) | 3,405 | 0 | “” | 0 | “” | 0 | “” | 3 | “” |
|  | Men | Total | 5,041 (100.0) | 89,543 | 40 | 45 (33-61) | 14 | 0.3 (0.2-0.5) | 36 | 0.9 (0.7-1.3) | 149 | 0.27 (0.19-0.37) |
|  |  | Age group at baseline, years |  |  |  |  |  |  |  |  |  |  |
|  |  | 55-59 | 2,274 (45.1) | 41,955 | 18 | 43 (27-68) | 6 | 0.3 (0.1-0.7) | 17 | 0.9 (0.6-1.5) | 61 | 0.29 (0.17-0.46) |
|  |  | 60-66 | 2,767 (54.9) | 47,587 | 22 | 46 (30-70) | 8 | 0.3 (0.2-0.7) | 19 | 0.9 (0.6-1.4) | 88 | 0.25 (0.16-0.38) |
|  |  | Endoscopist ADR ranking group^c,d^ |  |  |  |  |  |  |  |  |  |  |
|  |  | High | 2,307 (45.8) | 41,070 | 4 | 10 (4-26) | 2 | 0.1 (0.0-0.4) | 3 | 0.2 (0.1-0.5) | 69 | 0.06 (0.02-0.15) |
|  |  | Intermediate | 1,534 (30.4) | 27,738 | 14 | 50 (30-85) | 5 | 0.4 (0.2-0.9) | 14 | 1.1 (0.7-1.9) | 46 | 0.30 (0.17-0.51) |
|  |  | Low | 1,080 (21.4) | 18,722 | 20 | 107 (69-166) | 6 | 0.6 (0.3-1.4) | 18 | 2.2 (1.4-3.4) | 31 | 0.65 (0.39-1.00) |
|  |  | Bowel preparation quality^c,e^ |  |  |  |  |  |  |  |  |  |  |
|  |  | Excellent | 1,885 (37.4) | 34,135 | 14 | 41 (24-69) | 3 | 0.2 (0.1-0.5) | 12 | 0.8 (0.5-1.4) | 57 | 0.25 (0.13-0.41) |
|  |  | Good | 1,846 (36.6) | 32,727 | 11 | 34 (19-61) | 4 | 0.3 (0.1-0.7) | 10 | 0.7 (0.4-1.3) | 54 | 0.20 (0.10-0.36) |
|  |  | Adequate | 1,033 (20.5) | 17,610 | 12 | 68 (39-120) | 6 | 0.6 (0.3-1.4) | 12 | 1.5 (0.9-2.7) | 29 | 0.41 (0.21-0.71) |
|  |  | Poor | 39 (0.8) | 688 | 2 | 291 (73-1,162) | 1 | 3.0 (0.4-19.6) | 2 | 6.6 (1.7-24.1) | 1 | 1.71 (0.21-6.18) |
|  |  | Examination completeness^c,e^ |  |  |  |  |  |  |  |  |  |  |
|  |  | Complete | 4,797 (95.2) | 85,328 | 36 | 42 (30-58) | 12 | 0.3 (0.2-0.5) | 32 | 0.8 (0.6-1.2) | 142 | 0.25 (0.18-0.35) |
|  |  | Incomplete | 193 (3.8) | 3,286 | 4 | 122 (46-324) | 2 | 1.2 (0.3-4.8) | 4 | 2.7 (1.0-7.1) | 6 | 0.71 (0.19-1.82) |
|  |  | Adenoma size, mm^f^ |  |  |  |  |  |  |  |  |  |  |
|  |  | No adenomas | 3,025 (60.0) | 54,101 | 19 | 35 (22-55) | 4 | 0.1 (0.1-0.4) | 16 | 0.7 (0.4-1.1) | 90 | 0.21 (0.13-0.33) |
|  |  | <5 | 1,170 (23.2) | 21,160 | 10 | 47 (25-88) | 6 | 0.6 (0.3-1.3) | 9 | 0.9 (0.5-1.7) | 36 | 0.28 (0.13-0.52) |
|  |  | 5-9 | 846 (16.8) | 14,281 | 11 | 77 (43-139) | 4 | 0.6 (0.2-1.5) | 11 | 1.7 (0.9-3.0) | 24 | 0.46 (0.23-0.83) |
|  |  | No. of HPs |  |  |  |  |  |  |  |  |  |  |
|  |  | 0 | 1,578 (31.3) | 28,126 | 16 | 57 (35-93) | 9 | 0.6 (0.3-1.2) | 15 | 1.1 (0.7-1.9) | 47 | 0.34 (0.19-0.55) |
|  |  | 1-2 | 2,753 (54.6) | 49,476 | 23 | 46 (31-70) | 5 | 0.2 (0.1-0.5) | 20 | 0.9 (0.6-1.4) | 82 | 0.28 (0.18-0.42) |
|  |  | 3-19 | 710 (14.1) | 11,941 | 1 | 8 (1-59) | 0 | “” | 1 | 0.2 (0.0-1.6) | 20 | 0.05 (0.00-0.28) |
|  |  | HP size, mm^g^ |  |  |  |  |  |  |  |  |  |  |
|  |  | <5 | 2,690 (77.7) | 48,448 | 15 | 31 (19-51) | 3 | 0.1 (0.0-0.4) | 12 | 0.6 (0.3-1.0) | 81 | 0.19 (0.10-0.31) |
|  |  | 5-9 | 773 (22.3) | 12,969 | 9 | 69 (36-133) | 2 | 0.3 (0.1-1.2) | 9 | 1.6 (0.9-3.1) | 21 | 0.42 (0.19-0.80) |
|  |  | Location of HPs^h^ |  |  |  |  |  |  |  |  |  |  |
|  |  | RM/RS only | 1,643 (47.4) | 29,292 | 10 | 34 (18-63) | 2 | 0.1 (0.0-0.5) | 9 | 0.7 (0.4-1.3) | 49 | 0.20 (0.10-0.38) |
|  |  | Not confined to RM/RS | 1,820 (52.6) | 32,125 | 14 | 44 (26-74) | 3 | 0.2 (0.1-0.6) | 12 | 0.9 (0.5-1.6) | 53 | 0.26 (0.14-0.44) |
|  |  | Presence of adenomas and/or HPs |  |  |  |  |  |  |  |  |  |  |
|  |  | No adenomas or HPs^i^ | 145 (2.9) | 2,529 | 1 | 40 (6-281) | 1 | 0.8 (0.1-5.3) | 1 | 0.8 (0.1-5.3) | 4 | 0.24 (0.01-1.31) |
|  |  | Adenomas only | 1,433 (28.4) | 25,596 | 15 | 59 (35-97) | 8 | 0.6 (0.3-1.2) | 14 | 1.2 (0.7-2.0) | 43 | 0.35 (0.19-0.57) |
|  |  | HPs only | 2,880 (57.1) | 51,572 | 18 | 35 (22-55) | 3 | 0.1 (0.0-0.4) | 15 | 0.7 (0.4-1.1) | 86 | 0.21 (0.12-0.33) |
|  |  | Adenomas and HPs | 583 (11.6) | 9,845 | 6 | 61 (27-136) | 2 | 0.4 (0.1-1.6) | 6 | 1.3 (0.6-3.0) | 16 | 0.37 (0.14-0.81) |

ADR: adenoma detection rate. CI: confidence interval. CRC: colorectal cancer. HP: hyperplastic polyp. mm: millimetre. RM: rectum. RS: rectosigmoid. SIR: standardised incidence ratio.

^a^Participants in the low-risk group had polyps detected at baseline that did not meet the following high-risk criteria: an adenoma or HP ≥10mm, ≥3 adenomas, or an adenoma with villous/tubulovillous histology or high-grade dysplasia. Participants in the ‘no polyps’ group had no polyps detected at baseline.

^b^Included each participant’s follow-up time from start of time-at-risk, censored at any first surveillance visit.

^c^We do not present data for the ‘missing’ categories of endoscopist ADR ranking group, bowel preparation quality, or examination completeness due to low numbers of cancer cases (≤3) in the categories when stratifying by polyp group and sex. In the ‘no polyps’ group, there were 605, 125, and 44 women and 474, 120, and 44 men with missing values for endoscopist ADR ranking group, bowel preparation quality, and examination completeness, respectively. In the low-risk group, there were 95, 153, and 24 women and 120, 238, and 51 men with missing values for endoscopist ADR ranking group, bowel preparation quality, and examination completeness, respectively.

^d^Endoscopists were classified into high-, intermediate-, or low-detector ranking groups based on their ADR (with ADR calculated as the % of participants screened by the endoscopist who had ≥1 adenomas detected during screening).

^e^For participants who had flexible sigmoidoscopy (FS) at baseline and no index colonoscopy, bowel preparation quality and examination completeness were defined, respectively, according to the best quality preparation and most complete examination for an FS performed during the baseline visit. For those who had FS and an index colonoscopy at baseline, bowel preparation quality and examination completeness were defined, respectively, according to the best quality preparation and most complete examination for an FS or colonoscopy performed during the baseline visit.

^f^Defined according to the largest diameter recorded during the baseline visit.

^g^Defined according to the largest diameter recorded during the baseline visit; percentages were calculated using the number of participants with at least one baseline HP as the denominator.

^h^Percentages were calculated using the number of participants with at least one baseline HP as the denominator.

^i^Of participants in the low-risk group who had no adenomas or HPs, the majority had a baseline polyp reported to be an ‘assume polyp’; most were diagnosed as adenomas at endoscopy sighting but were lost after removal, preventing pathological confirmation of polyp type.

**Supplementary Table 5. Proximal cancer incidence without surveillance and age-sex-standardised incidence ratios in women and men in the ‘no polyps’ and low-risk groups, by baseline patient, procedural, and polyp characteristics (n=37,954)**

| **Polyp group^a^** | **Sex** | **Baseline characteristic** | **n (%)** | **No. of person-years^b^** | **Total no. of cases** | **Incidence rate per 100,000 person-years (95%CI)** | **Follow-up** | |  | | **Standardisation** | |
| --- | --- | --- | --- | --- | --- | --- | --- | --- | --- | --- | --- | --- |
|  |  |  |  |  |  |  | 10 years | | 20 years | |  |  |
|  |  |  |  |  |  |  | No. of cases | Cumulative incidence, % (95%CI) | No. of cases | Cumulative incidence, % (95%CI) | No. of expected cases | SIR (95%CI) |
| No polyps | Women | Total | 16,059 (100.0) | 313,279 | 212 | 68 (59-77) | 60 | 0.4 (0.3-0.5) | 184 | 1.3 (1.1-1.5) | 198 | 1.07 (0.93-1.22) |
|  |  | Age group at baseline, years |  |  |  |  |  |  |  |  |  |  |
|  |  | 55-59 | 7,402 (46.1) | 147,762 | 87 | 59 (48-73) | 21 | 0.3 (0.2-0.5) | 72 | 1.1 (0.9-1.4) | 78 | 1.12 (0.90-1.38) |
|  |  | 60-66 | 8,657 (53.9) | 165,517 | 125 | 76 (63-90) | 39 | 0.5 (0.3-0.6) | 112 | 1.5 (1.2-1.8) | 120 | 1.04 (0.86-1.24) |
|  |  | Endoscopist ADR ranking group^c,d^ |  |  |  |  |  |  |  |  |  |  |
|  |  | High | 5,484 (34.1) | 107,155 | 68 | 63 (50-80) | 17 | 0.3 (0.2-0.5) | 59 | 1.2 (0.9-1.6) | 68 | 1.00 (0.78-1.27) |
|  |  | Intermediate | 4,827 (30.1) | 94,721 | 67 | 71 (56-90) | 20 | 0.4 (0.3-0.7) | 57 | 1.3 (1.0-1.7) | 60 | 1.12 (0.87-1.42) |
|  |  | Low | 5,143 (32.0) | 99,240 | 72 | 73 (58-91) | 22 | 0.4 (0.3-0.7) | 63 | 1.4 (1.1-1.8) | 62 | 1.16 (0.91-1.46) |
|  |  | Bowel preparation quality^c,e^ |  |  |  |  |  |  |  |  |  |  |
|  |  | Excellent | 7,194 (44.8) | 141,930 | 96 | 68 (55-83) | 30 | 0.4 (0.3-0.6) | 81 | 1.3 (1.0-1.6) | 90 | 1.07 (0.87-1.31) |
|  |  | Good | 5,367 (33.4) | 104,279 | 68 | 65 (51-83) | 17 | 0.3 (0.2-0.5) | 58 | 1.2 (0.9-1.6) | 66 | 1.03 (0.80-1.31) |
|  |  | Adequate | 2,954 (18.4) | 56,778 | 34 | 60 (43-84) | 8 | 0.3 (0.1-0.6) | 32 | 1.3 (0.9-1.8) | 36 | 0.94 (0.65-1.32) |
|  |  | Poor | 419 (2.6) | 7,814 | 11 | 141 (78-254) | 4 | 1.0 (0.4-2.7) | 11 | 3.1 (1.7-5.5) | 5 | 2.25 (1.12-4.03) |
|  |  | Examination completeness^c,e^ |  |  |  |  |  |  |  |  |  |  |
|  |  | Complete | 13,447 (83.7) | 263,653 | 171 | 65 (56-75) | 43 | 0.3 (0.2-0.4) | 146 | 1.2 (1.0-1.4) | 167 | 1.03 (0.88-1.19) |
|  |  | Incomplete | 2,568 (16.0) | 48,785 | 41 | 84 (62-114) | 17 | 0.7 (0.4-1.1) | 38 | 1.7 (1.2-2.3) | 31 | 1.32 (0.95-1.79) |
|  | Men | Total | 13,733 (100.0) | 253,445 | 154 | 61 (52-71) | 47 | 0.4 (0.3-0.5) | 137 | 1.2 (1.0-1.4) | 204 | 0.75 (0.64-0.88) |
|  |  | Age group at baseline, years |  |  |  |  |  |  |  |  |  |  |
|  |  | 55-59 | 6,468 (47.1) | 124,401 | 68 | 55 (43-69) | 19 | 0.3 (0.2-0.5) | 57 | 1.0 (0.8-1.3) | 84 | 0.81 (0.63-1.03) |
|  |  | 60-66 | 7,265 (52.9) | 129,043 | 86 | 67 (54-82) | 28 | 0.4 (0.3-0.6) | 80 | 1.4 (1.1-1.7) | 121 | 0.71 (0.57-0.88) |
|  |  | Endoscopist ADR ranking group^c,d^ |  |  |  |  |  |  |  |  |  |  |
|  |  | High | 4,486 (32.7) | 82,774 | 60 | 72 (56-93) | 21 | 0.5 (0.3-0.8) | 53 | 1.4 (1.1-1.8) | 67 | 0.90 (0.69-1.16) |
|  |  | Intermediate | 4,145 (30.2) | 78,110 | 32 | 41 (29-58) | 6 | 0.2 (0.1-0.3) | 28 | 0.8 (0.6-1.2) | 64 | 0.50 (0.34-0.71) |
|  |  | Low | 4,628 (33.7) | 83,621 | 55 | 66 (51-86) | 17 | 0.4 (0.2-0.6) | 49 | 1.3 (1.0-1.7) | 66 | 0.83 (0.62-1.08) |
|  |  | Bowel preparation quality^c,e^ |  |  |  |  |  |  |  |  |  |  |
|  |  | Excellent | 5,573 (40.6) | 104,926 | 55 | 52 (40-68) | 11 | 0.2 (0.1-0.4) | 49 | 1.1 (0.8-1.4) | 85 | 0.65 (0.49-0.85) |
|  |  | Good | 4,679 (34.1) | 85,661 | 53 | 62 (47-81) | 24 | 0.5 (0.4-0.8) | 47 | 1.2 (0.9-1.6) | 69 | 0.77 (0.58-1.01) |
|  |  | Adequate | 3,108 (22.6) | 56,269 | 40 | 71 (52-97) | 11 | 0.4 (0.2-0.7) | 35 | 1.4 (1.0-1.9) | 46 | 0.87 (0.62-1.19) |
|  |  | Poor | 253 (1.8) | 4,367 | 4 | 92 (34-244) | 1 | 0.4 (0.1-2.9) | 4 | 2.0 (0.7-5.3) | 4 | 1.12 (0.31-2.88) |
|  |  | Examination completeness^c,e^ |  |  |  |  |  |  |  |  |  |  |
|  |  | Complete | 12,781 (93.1) | 236,749 | 150 | 63 (54-74) | 46 | 0.4 (0.3-0.5) | 133 | 1.2 (1.0-1.5) | 191 | 0.79 (0.67-0.92) |
|  |  | Incomplete | 908 (6.6) | 15,923 | 3 | 19 (6-58) | 1 | 0.1 (0.0-0.8) | 3 | 0.4 (0.1-1.2) | 13 | 0.23 (0.05-0.67) |
| Low-risk | Women | Total | 3,121 (100.0) | 58,710 | 82 | 140 (112-173) | 23 | 0.8 (0.5-1.2) | 74 | 2.8 (2.2-3.5) | 37 | 2.22 (1.77-2.76) |
|  |  | Age group at baseline, years |  |  |  |  |  |  |  |  |  |  |
|  |  | 55-59 | 1,379 (44.2) | 26,678 | 32 | 120 (85-169) | 9 | 0.7 (0.4-1.3) | 26 | 2.1 (1.5-3.1) | 14 | 2.31 (1.58-3.26) |
|  |  | 60-66 | 1,742 (55.8) | 32,033 | 50 | 156 (118-206) | 14 | 0.9 (0.5-1.4) | 48 | 3.3 (2.5-4.3) | 23 | 2.17 (1.61-2.86) |
|  |  | Endoscopist ADR ranking group^c,d^ |  |  |  |  |  |  |  |  |  |  |
|  |  | High | 1,513 (48.5) | 28,148 | 29 | 103 (72-148) | 8 | 0.6 (0.3-1.1) | 26 | 2.0 (1.4-2.9) | 18 | 1.65 (1.11-2.37) |
|  |  | Intermediate | 885 (28.4) | 16,967 | 35 | 206 (148-287) | 11 | 1.3 (0.7-2.3) | 31 | 4.0 (2.8-5.6) | 11 | 3.23 (2.25-4.49) |
|  |  | Low | 628 (20.1) | 11,891 | 17 | 143 (89-230) | 3 | 0.5 (0.2-1.6) | 16 | 3.1 (1.9-4.9) | 7 | 2.29 (1.34-3.67) |
|  |  | Bowel preparation quality^c,e^ |  |  |  |  |  |  |  |  |  |  |
|  |  | Excellent | 1,216 (39.0) | 23,292 | 34 | 146 (104-204) | 9 | 0.8 (0.4-1.5) | 30 | 2.8 (2.0-4.0) | 15 | 2.32 (1.60-3.24) |
|  |  | Good | 1,145 (36.7) | 21,467 | 33 | 154 (109-216) | 9 | 0.8 (0.4-1.6) | 31 | 3.1 (2.2-4.4) | 14 | 2.44 (1.68-3.43) |
|  |  | Adequate | 577 (18.5) | 10,497 | 8 | 76 (38-152) | 2 | 0.4 (0.1-1.4) | 7 | 1.5 (0.7-3.0) | 7 | 1.23 (0.53-2.42) |
|  |  | Poor | 30 (1.0) | 584 | 1 | 171 (24-1,216) | 1 | 3.3 (0.5-21.4) | 1 | 3.3 (0.5-21.4) | 0 | 2.59 (0.07-14.42) |
|  |  | Examination completeness^c,e^ |  |  |  |  |  |  |  |  |  |  |
|  |  | Complete | 2,671 (85.6) | 50,421 | 77 | 153 (122-191) | 23 | 0.9 (0.6-1.4) | 70 | 3.0 (2.4-3.8) | 32 | 2.43 (1.92-3.04) |
|  |  | Incomplete | 426 (13.6) | 7,818 | 5 | 64 (27-154) | 0 | “” | 4 | 1.3 (0.5-3.4) | 5 | 1.01 (0.33-2.36) |
|  |  | Adenoma size, mm^f^ |  |  |  |  |  |  |  |  |  |  |
|  |  | No adenomas | 2,039 (65.3) | 38,387 | 51 | 133 (101-175) | 15 | 0.8 (0.5-1.3) | 45 | 2.6 (1.9-3.5) | 24 | 2.12 (1.58-2.79) |
|  |  | <5 | 667 (21.4) | 12,683 | 16 | 126 (77-206) | 5 | 0.8 (0.3-1.9) | 16 | 2.7 (1.7-4.4) | 8 | 2.00 (1.15-3.26) |
|  |  | 5-9 | 415 (13.3) | 7,640 | 15 | 196 (118-326) | 3 | 0.8 (0.3-2.4) | 13 | 3.6 (2.1-6.2) | 5 | 3.08 (1.72-5.08) |
|  |  | No. of HPs |  |  |  |  |  |  |  |  |  |  |
|  |  | 0 | 1,003 (32.1) | 19,114 | 23 | 120 (80-181) | 4 | 0.4 (0.2-1.1) | 21 | 2.4 (1.6-3.7) | 12 | 1.88 (1.19-2.82) |
|  |  | 1-2 | 1,780 (57.0) | 33,786 | 46 | 136 (102-182) | 14 | 0.8 (0.5-1.4) | 41 | 2.7 (2.0-3.6) | 21 | 2.18 (1.59-2.90) |
|  |  | 3-19 | 338 (10.8) | 5,811 | 13 | 224 (130-385) | 5 | 1.6 (0.7-3.9) | 12 | 4.3 (2.5-7.5) | 4 | 3.67 (1.96-6.28) |
|  |  | HP size, mm^g^ |  |  |  |  |  |  |  |  |  |  |
|  |  | <5 | 1,748 (82.5) | 33,059 | 45 | 136 (102-182) | 13 | 0.8 (0.5-1.3) | 40 | 2.6 (1.9-3.5) | 21 | 2.17 (1.59-2.91) |
|  |  | 5-9 | 370 (17.5) | 6,537 | 14 | 214 (127-362) | 6 | 1.8 (0.8-3.9) | 13 | 4.5 (2.6-7.7) | 4 | 3.52 (1.92-5.90) |
|  |  | Location of HPs^h^ |  |  |  |  |  |  |  |  |  |  |
|  |  | RM/RS only | 1,130 (53.4) | 21,251 | 23 | 108 (72-163) | 5 | 0.5 (0.2-1.1) | 19 | 2.0 (1.3-3.1) | 13 | 1.74 (1.10-2.61) |
|  |  | Not confined to RM/RS | 988 (46.6) | 18,346 | 36 | 196 (142-272) | 14 | 1.5 (0.9-2.5) | 34 | 4.0 (2.9-5.6) | 11 | 3.15 (2.20-4.35) |
|  |  | Presence of adenomas and/or HPs |  |  |  |  |  |  |  |  |  |  |
|  |  | No adenomas or HPs^i^ | 117 (3.7) | 2,196 | 0 | “” | 0 | “” | 0 | “” | 1 | “” |
|  |  | Adenomas only | 886 (28.4) | 16,918 | 23 | 136 (90-205) | 4 | 0.5 (0.2-1.3) | 21 | 2.7 (1.8-4.1) | 11 | 2.13 (1.35-3.20) |
|  |  | HPs only | 1,922 (61.6) | 36,191 | 51 | 141 (107-185) | 15 | 0.8 (0.5-1.4) | 45 | 2.7 (2.1-3.7) | 23 | 2.25 (1.68-2.96) |
|  |  | Adenomas and HPs | 196 (6.3) | 3,405 | 8 | 235 (118-470) | 4 | 2.2 (0.8-5.9) | 8 | 4.7 (2.4-9.3) | 2 | 3.89 (1.68-7.67) |
|  | Men | Total | 5,041 (100.0) | 89,543 | 88 | 98 (80-121) | 24 | 0.5 (0.4-0.8) | 78 | 2.0 (1.6-2.4) | 72 | 1.22 (0.98-1.51) |
|  |  | Age group at baseline, years |  |  |  |  |  |  |  |  |  |  |
|  |  | 55-59 | 2,274 (45.1) | 41,955 | 38 | 91 (66-124) | 8 | 0.4 (0.2-0.8) | 34 | 1.8 (1.3-2.5) | 28 | 1.36 (0.96-1.87) |
|  |  | 60-66 | 2,767 (54.9) | 47,587 | 50 | 105 (80-139) | 16 | 0.6 (0.4-1.0) | 44 | 2.1 (1.5-2.8) | 44 | 1.14 (0.84-1.50) |
|  |  | Endoscopist ADR ranking group^c,d^ |  |  |  |  |  |  |  |  |  |  |
|  |  | High | 2,307 (45.8) | 41,070 | 42 | 102 (76-138) | 10 | 0.5 (0.3-0.9) | 38 | 2.1 (1.5-2.8) | 33 | 1.27 (0.92-1.72) |
|  |  | Intermediate | 1,534 (30.4) | 27,738 | 28 | 101 (70-146) | 8 | 0.6 (0.3-1.1) | 25 | 2.0 (1.4-3.0) | 22 | 1.25 (0.83-1.81) |
|  |  | Low | 1,080 (21.4) | 18,722 | 17 | 91 (56-146) | 5 | 0.5 (0.2-1.2) | 14 | 1.7 (1.0-2.8) | 15 | 1.14 (0.67-1.83) |
|  |  | Bowel preparation quality^c,e^ |  |  |  |  |  |  |  |  |  |  |
|  |  | Excellent | 1,885 (37.4) | 34,135 | 36 | 105 (76-146) | 7 | 0.4 (0.2-0.9) | 32 | 2.2 (1.5-3.0) | 27 | 1.32 (0.92-1.82) |
|  |  | Good | 1,846 (36.6) | 32,727 | 36 | 110 (79-153) | 12 | 0.7 (0.4-1.2) | 30 | 2.0 (1.4-2.8) | 26 | 1.37 (0.96-1.90) |
|  |  | Adequate | 1,033 (20.5) | 17,610 | 12 | 68 (39-120) | 4 | 0.4 (0.2-1.1) | 12 | 1.6 (0.9-2.9) | 14 | 0.85 (0.44-1.48) |
|  |  | Poor | 39 (0.8) | 688 | 0 | “” | 0 | “” | 0 | “” | 1 | “” |
|  |  | Examination completeness^c,e^ |  |  |  |  |  |  |  |  |  |  |
|  |  | Complete | 4,797 (95.2) | 85,328 | 84 | 98 (79-122) | 22 | 0.5 (0.3-0.8) | 74 | 1.9 (1.5-2.4) | 68 | 1.23 (0.98-1.52) |
|  |  | Incomplete | 193 (3.8) | 3,286 | 2 | 61 (15-243) | 0 | “” | 2 | 1.7 (0.4-6.6) | 3 | 0.73 (0.09-2.64) |
|  |  | Adenoma size, mm^f^ |  |  |  |  |  |  |  |  |  |  |
|  |  | No adenomas | 3,025 (60.0) | 54,101 | 45 | 83 (62-111) | 15 | 0.5 (0.3-0.9) | 39 | 1.6 (1.2-2.2) | 43 | 1.04 (0.76-1.39) |
|  |  | <5 | 1,170 (23.2) | 21,160 | 19 | 90 (57-141) | 4 | 0.4 (0.1-1.0) | 16 | 1.7 (1.0-2.8) | 17 | 1.10 (0.66-1.72) |
|  |  | 5-9 | 846 (16.8) | 14,281 | 24 | 168 (113-251) | 5 | 0.7 (0.3-1.7) | 23 | 3.7 (2.5-5.6) | 11 | 2.10 (1.35-3.13) |
|  |  | No. of HPs |  |  |  |  |  |  |  |  |  |  |
|  |  | 0 | 1,578 (31.3) | 28,126 | 30 | 107 (75-153) | 9 | 0.6 (0.3-1.2) | 29 | 2.3 (1.6-3.3) | 23 | 1.31 (0.88-1.87) |
|  |  | 1-2 | 2,753 (54.6) | 49,476 | 48 | 97 (73-129) | 11 | 0.4 (0.2-0.8) | 41 | 1.8 (1.4-2.5) | 40 | 1.21 (0.89-1.61) |
|  |  | 3-19 | 710 (14.1) | 11,941 | 10 | 84 (45-156) | 4 | 0.6 (0.2-1.6) | 8 | 1.5 (0.7-3.0) | 9 | 1.07 (0.51-1.96) |
|  |  | HP size, mm^g^ |  |  |  |  |  |  |  |  |  |  |
|  |  | <5 | 2,690 (77.7) | 48,448 | 42 | 87 (64-117) | 9 | 0.4 (0.2-0.7) | 34 | 1.6 (1.1-2.2) | 39 | 1.08 (0.78-1.46) |
|  |  | 5-9 | 773 (22.3) | 12,969 | 16 | 123 (76-201) | 6 | 0.8 (0.4-1.9) | 15 | 2.5 (1.5-4.2) | 10 | 1.57 (0.90-2.55) |
|  |  | Location of HPs^h^ |  |  |  |  |  |  |  |  |  |  |
|  |  | RM/RS only | 1,643 (47.4) | 29,292 | 25 | 85 (58-126) | 6 | 0.4 (0.2-0.9) | 20 | 1.5 (1.0-2.3) | 24 | 1.06 (0.69-1.57) |
|  |  | Not confined to RM/RS | 1,820 (52.6) | 32,125 | 33 | 103 (73-144) | 9 | 0.5 (0.3-1.0) | 29 | 2.0 (1.4-2.9) | 25 | 1.30 (0.89-1.82) |
|  |  | Presence of adenomas and/or HPs |  |  |  |  |  |  |  |  |  |  |
|  |  | No adenomas or HPs^i^ | 145 (2.9) | 2,529 | 3 | 119 (38-368) | 2 | 1.5 (0.4-5.8) | 3 | 2.5 (0.8-7.5) | 2 | 1.46 (0.30-4.27) |
|  |  | Adenomas only | 1,433 (28.4) | 25,596 | 27 | 105 (72-154) | 7 | 0.6 (0.3-1.2) | 26 | 2.3 (1.6-3.4) | 21 | 1.29 (0.85-1.88) |
|  |  | HPs only | 2,880 (57.1) | 51,572 | 42 | 81 (60-110) | 13 | 0.5 (0.3-0.8) | 36 | 1.5 (1.1-2.1) | 41 | 1.02 (0.73-1.38) |
|  |  | Adenomas and HPs | 583 (11.6) | 9,845 | 16 | 163 (100-265) | 2 | 0.4 (0.1-1.6) | 13 | 3.0 (1.8-5.2) | 8 | 2.07 (1.18-3.35) |

ADR: adenoma detection rate. CI: confidence interval. CRC: colorectal cancer. HP: hyperplastic polyp. mm: millimetre. RM: rectum. RS: rectosigmoid. SIR: standardised incidence ratio.

^a^Participants in the low-risk group had polyps detected at baseline that did not meet the following high-risk criteria: an adenoma or HP ≥10mm, ≥3 adenomas, or an adenoma with villous/tubulovillous histology or high-grade dysplasia. Participants in the ‘no polyps’ group had no polyps detected at baseline.

^b^Included each participant’s follow-up time from start of time-at-risk, censored at any first surveillance visit.

^c^We do not present data for the ‘missing’ categories of endoscopist ADR ranking group, bowel preparation quality, or examination completeness due to low numbers of cancer cases (≤7) in the categories when stratifying by polyp group and sex. In the ‘no polyps’ group, there were 605, 125, and 44 women and 474, 120, and 44 men with missing values for endoscopist ADR ranking group, bowel preparation quality, and examination completeness, respectively. In the low-risk group, there were 95, 153, and 24 women and 120, 238, and 51 men with missing values for endoscopist ADR ranking group, bowel preparation quality, and examination completeness, respectively.

^d^Endoscopists were classified into high-, intermediate-, or low-detector ranking groups based on their ADR (with ADR calculated as the % of participants screened by the endoscopist who had ≥1 adenomas detected during screening).

^e^For participants who had flexible sigmoidoscopy (FS) at baseline and no index colonoscopy, bowel preparation quality and examination completeness were defined, respectively, according to the best quality preparation and most complete examination for an FS performed during the baseline visit. For those who had FS and an index colonoscopy at baseline, bowel preparation quality and examination completeness were defined, respectively, according to the best quality preparation and most complete examination for an FS or colonoscopy performed during the baseline visit.

^f^Defined according to the largest diameter recorded during the baseline visit.

^g^Defined according to the largest diameter recorded during the baseline visit; percentages were calculated using the number of participants with at least one baseline HP as the denominator.

^h^Percentages were calculated using the number of participants with at least one baseline HP as the denominator.

^i^Of participants in the low-risk group who had no adenomas or HPs, the majority had a baseline polyp reported to be an ‘assume polyp’; most were diagnosed as adenomas at endoscopy sighting but were lost after removal, preventing pathological confirmation of polyp type.
